# Mechanistic Model of *Rothia mucilaginosa* Adaptation toward Persistence in the CF Lung, Based on a Genome Reconstructed from Metagenomic Data

**DOI:** 10.1371/journal.pone.0064285

**Published:** 2013-05-30

**Authors:** Yan Wei Lim, Robert Schmieder, Matthew Haynes, Mike Furlan, T. David Matthews, Katrine Whiteson, Stephen J. Poole, Christopher S. Hayes, David A. Low, Heather Maughan, Robert Edwards, Douglas Conrad, Forest Rohwer

**Affiliations:** 1 Department of Biology, San Diego State University, San Diego, California, United States of America; 2 Computational Science Research Center, San Diego State University, San Diego, California, United States of America; 3 Department of Molecular, Cellular, and Developmental Biology, University of California Santa Barbara, Santa Barbara, California, United States of America; 4 Biomolecular Science and Engineering Program, University of California Santa Barbara, Santa Barbara, California, United States of America; 5 Ronin Institute, Montclair, New Jersey, United States of America; 6 Mathematics and Computer Science Division, Argonne National Laboratory, Argonne, Illinois, United States of America; 7 Department of Medicine, University of California San Diego, La Jolla, California, United States of America; Abramson Research Center, United States of America

## Abstract

The impaired mucociliary clearance in individuals with Cystic Fibrosis (CF) enables opportunistic pathogens to colonize CF lungs. Here we show that *Rothia mucilaginosa* is a common CF opportunist that was present in 83% of our patient cohort, almost as prevalent as *Pseudomonas aeruginosa* (89%). Sequencing of lung microbial metagenomes identified unique *R. mucilaginosa* strains in each patient, presumably due to evolution within the lung. The *de novo* assembly of a near-complete *R. mucilaginosa* (CF1E) genome illuminated a number of potential physiological adaptations to the CF lung, including antibiotic resistance, utilization of extracellular lactate, and modification of the type I restriction-modification system. Metabolic characteristics predicted from the metagenomes suggested *R. mucilaginosa* have adapted to live within the microaerophilic surface of the mucus layer in CF lungs. The results also highlight the remarkable evolutionary and ecological similarities of many CF pathogens; further examination of these similarities has the potential to guide patient care and treatment.

## Introduction

Cystic fibrosis (CF) is a genetic disease caused by mutation of the cystic fibrosis transmembrane conductance regulator (CFTR) gene [1]. In CF lungs, the defective CFTR protein affects trans-epithelial ion transport and consequently leads to the accumulation of thick and static mucus. The resultant hypoxic microenvironment encourages the colonization of opportunistic microbes, viruses, and fungi (reviewed in [2]), causing acute and chronic infection. A few of the most commonly isolated pathogens are *Pseudomonas aeruginosa*, *Staphylococcus aureus*, *Haemophilus influenzae,* and *Burkholderia cepacia*. However, an increasing number of microbial species have been detected in the CF airway using culture-independent methods such as metagenomic sequencing [3]–[7]. Metagenomics is a powerful approach that has been used to successfully characterize the microbial and viral communities in CF individuals [8]–[12]. These types of studies have illuminated the complexity of microbial and viral communities, captured the vast diversity of functions encoded by these organisms, and have been used to trace the evolution of whole genomes [13], [14].

Previous sequencing of CF metagenomes revealed the presence of *Rothia mucilaginosa* at relatively high abundances in most patients [12]. *R. mucilaginosa* was first isolated from milk in 1900 as *Micrococcus mucilaginosus* [15]. It was later re-isolated and further studied by Bergan *et al.* in 1970 [16], and renamed *Stomatococcus mucilaginosus* in 1982 based on its 16S rDNA and biochemical characteristics [17]. A recent study comparing *S. mucilaginosus* to *Rothia dentocariosa* and another unknown species (later known as *Rothia nasimurium*) led to the reclassification of *S. mucilaginosus* as *R. mucilaginosa* [18].

*R. mucilaginosa* is an encapsulated, Gram-positive non-motile coccus (arranged in clusters) belonging to the phylum Actinobacteria. It has variable catalase activity, reduces nitrate, and hydrolyses aesculin [17]–[19]. It is a facultative anaerobe commonly found in the human oral cavity and upper respiratory tract [16], [20], [21], and occasionally the gastrointestinal tract [22], small intestinal epithelial lining [23], tongue [24], [25], teeth [26], [27], colostrum [28], breast milk [29], and dental plaques [30], [31]. Although *R. mucilaginosa* is commonly regarded as normal flora of the oral cavity and upper respiratory tract, its association with a wide range of diseases (Table S1) highlights its potential as an opportunistic pathogen, especially in immuno-compromised patients [32].

At the genus level, *Rothia* has been reported by Tunney *et al.* [33] as an aerobic species that can be isolated from CF sputum and pediatric bronchoalveolar lavage (BAL) samples. It has also been detected under anaerobic culturing conditions and via 16S rRNA gene surveys [33]. Typically *R. dentocariosa* was the main species identified in these studies [34]. In addition, Bittar *et al.* [5] and Guss *et al.* [7] have characterized *R. mucilaginosa* as a “newly” emerging CF pathogen. Even so, *R. mucilaginosa* is usually treated as part of the normal oral microbiota in the clinical lab. As a result, the presence of *R. mucilaginosa* in CF lungs may be under-reported and the significance of infection is underestimated.

Here we confirm that *R. mucilaginosa* is present and metabolically active in the lungs of CF patients. Comparisons with a non-CF reference genome revealed the presence of unique *R. mucilaginosa* strains in each patient. A near-complete genome was reconstructed from the metagenomic reads of one patient; comparison of these sequence data with a non-CF reference genome enabled the identification of unique genomic features that may have facilitated adaptation to the lung environment.

## Results and Discussion

Mutations in the CFTR locus affect proper ion transport in lung epithelial cells, impairing the clearance of mucus in the airways and encouraging microbial colonization and persistence. Until recently, most laboratory and clinical microbiology only focused on a few pathogens, particularly *P. aeruginosa*. This “one mutation, one pathogen” model of the CF ecosystem is being replaced by a “polyphysiology, polymicrobial” view that is expected to improve treatment until gene-therapy is able to fix the underlying genetic cause.

An important step in understanding the CF lung ecosystem, with the ultimate goal of eliminating microbes or altering their pathogenicity, is to determine which microbes are present and the ways in which their survival depends on local chemistry. An initial step in this direction is to use metagenomic and metatranscriptomic data previously generated from microbes and viruses present in CF sputum samples [12]. Metagenomic data provide information on which microbes and viruses are present, and their metabolic capabilities, while metatranscriptomic data provide information on which organisms are metabolically active [12]. Such metabolism data will enable predictions of local lung chemistry that may impose patient-specific selective pressures on the microbes. Data from eighteen microbiomes existing in six CF patients with different health statuses were analyzed here (Supporting Information S1; Table S2).

**Figure 1:**
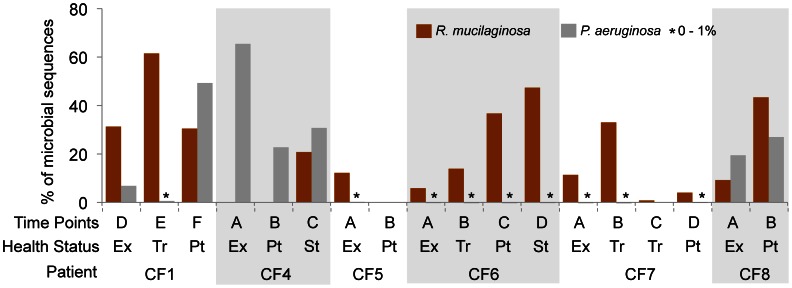
Prevalence of *Rothia mucilaginosa*, and the prototypical pathogen *Pseudomonas aeruginosa*, in eighteen microbiomes from six CF patients. Patients were sampled at times of differing health status (Table S1; [12]). Ex: Exacerbation; Tr: On treatment; Pt: Post treatment; St: Stable; *:present in <1% of the microbiome.

The metagenomic data showed that 15/18 (83%) sputa contained *R. mucilaginosa* and 16/18 (89%) sputa contained the common CF pathogen *P. aeruginosa*. Although *P. aeruginosa* was present in a greater number of samples, its abundance was lower than that of *R. mucilaginosa* in 11 of the 14 samples where these species co-existed. Both species abundances ranged from 1% to 62% (Figure 1). The relative percentages of these two opportunistic pathogens varied between patients and within the same patient as their health status changed. The results show no obvious pattern of synergy or competition between the two pathogens.

A health status of ‘Ex’ (for exacerbation) indicates a stark decline in lung function that is typically treated with intravenous antibiotics. Thus, between a health status of ‘Ex’ and ‘Tr’, patients will have been given antibiotics in addition to those that are often prescribed as continued therapy. The abundance data in Figure 1 indicate that these exacerbation-associated antibiotic treatments did little to permanently exclude *R. mucilaginosa* from CF lung communities. Patients CF1, CF4, CF6, CF7, and CF8 all had appreciable abundances of *R. mucilaginosa* by the last sampling time point. Only the *R. mucilaginosa* population in CF5 did not recover from antibiotic treatment by the last sampling time point; however, as this patient was only followed for 21 days (compared to 17–58 for the other patients), it is possible *R. mucilaginosa* could still rebound from antibiotic treatment. These results indicate that *R. mucilaginosa* is able to survive the typical CF antibiotic treatment, as is the main CF pathogen *P. aeruginosa*.

### *R. mucilaginosa* is Present in CF Lung Explants and Metabolically Active

The presence of *R. mucilaginosa* DNA in sputum samples (as detected by metagenome sequencing) could be explained by its abundance in the oral cavity and subsequent contamination of the sputum during collection. However, this is unlikely for several reasons. Previous studies have indicated little contamination of sampled sputa with oral inhabitants [35], [36], and the presence of *Rothia* has been confirmed in CF lungs [37]. Examination of lung tissue samples was the best way to definitively determine the presence of *R. mucilaginosa* in CF lungs from our cohort. Between 5 and 6 lung tissue sections from explanted lungs of each of four transplant patients were screened for *Rothia*-related microbes using 16S rDNA targeted PCR and sequencing. One out of the four patients was positive for *Rothia* (Supporting Information S3), indicating this bacterium is indeed present within lung airways. Unfortunately this patient was not available for the metagenome sequencing. The *R. mucilaginosa* population present in the oral cavity may serve as a reservoir and “stepping stone” for lower respiratory infection, as described in many respiratory chronic infections such as CF and chronic obstructive pulmonary disease [38].

The presence of *R. mucilaginosa* DNA in sputum or lung tissues does not necessarily indicate this bacterium is metabolically active in the lung environment. Examination of a metatranscriptome dataset indicated that mRNAs and rRNAs are being produced by *Rothia* species (Supporting Information S2), which suggests this bacterium is metabolically active in the CF lung.

### Genetic Differences of *R. mucilaginosa* between Patients

Longitudinal studies of *P. aeruginosa* [39], *Burkholderia dolosa* [40], and *Staphylococcus aureus* [41] within and between CF patients have shown evolutionary adaption to the CF lung. Here, we define adaptation as a process where mutations that alter pathogen behavior (in this case, metabolism) become fixed in response to specific environmental pressures, e.g. the availability of nutrients, oxygen, or redox potential. The power of the metagenomic data is in its ability to uncover the genetic mutations underlying these adaptations, that occur over long periods of selection. Characterizing these mutations thus enables us to infer which selection pressures are strongest in the CF lung, whether they be the dynamic lung physiology, immune system surveillance, and/or antibiotic treatment.

**Figure 2:**
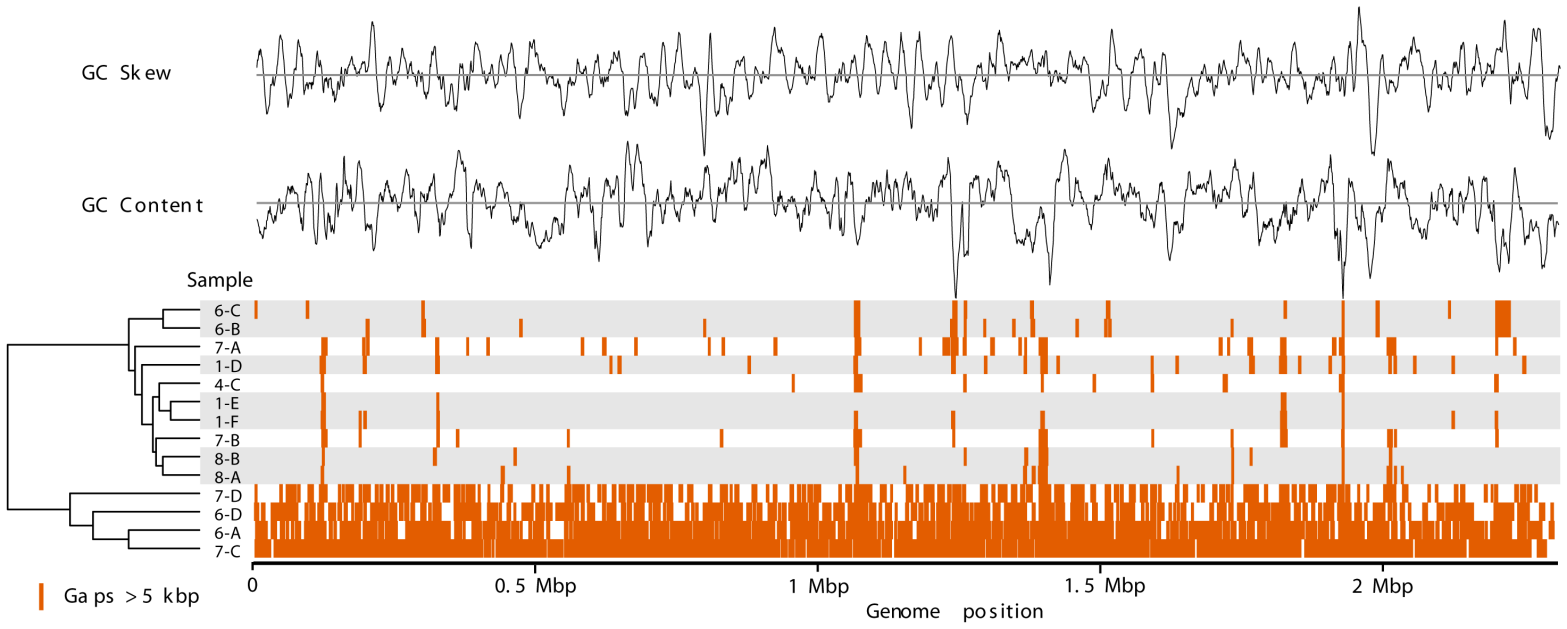
Hierarchical clustering of the sample based on gap patterns, which correspond to regions of the reference genome *R. mucilaginosa* DY-18 that were not represented by any metagenomic reads. BWA mapping was used and gaps were identified in a 1 kbp stepwise window. Only gaps ≥5 kbp were plotted. Exact coordinates and annotations for the gaps are in Table S3. The clade composed of 7-D, 6-D, 6-A, and 7-C appears to be lacking the majority of the reference genome, due to the low sequence coverage of *R. mucilaginosa* in these metagenomes.

We found evidence for similar evolutionary adaptation in *R. mucilaginosa*. The metagenomic sequences from each sample were mapped separately against the reference genome *R. mucilaginosa* DY-18, (GI: 283457089; originally isolated from persistent apical periodontitis lesions [42]. As shown in Figure 2, the mapped sequences reveal gaps where portions of the reference genome sequence were not covered by metagenomic reads (i.e., were absent) in the CF-derived datasets (gap patterns >5 kbp shown in Figure 2; Table S3). The out-group in Figure 2 is due to low coverage of *R. mucilaginosa* reads in the metagenomes of these patient samples (<1X coverage; Table S4). Most of the gaps occurred in regions of low GC content (Figure 2), which most likely represent genes acquired by DY-18 via horizontal gene transfer [43].

The gap patterns were most different between patients, indicating unique *R. mucilaginosa* strains exist in each patient. Within each patient, differences in gap patterns between time points were less numerous, but their existence indicates that the genome of *R. mucilaginosa* has been evolving independently in each patient. Combined with similar findings for *P. aeruginosa* [39] and *S. aureus* [41], this suggests that essentially every CF patient harbors a unique strain of *R. mucilaginosa* that evolves in the lung. If each strain also has a unique antibiotic resistance profile, then CF treatment will need to be tailored to the particular strain present in each patient.

### Characteristics of CF1E Genome Scaffold

**Figure 3:**
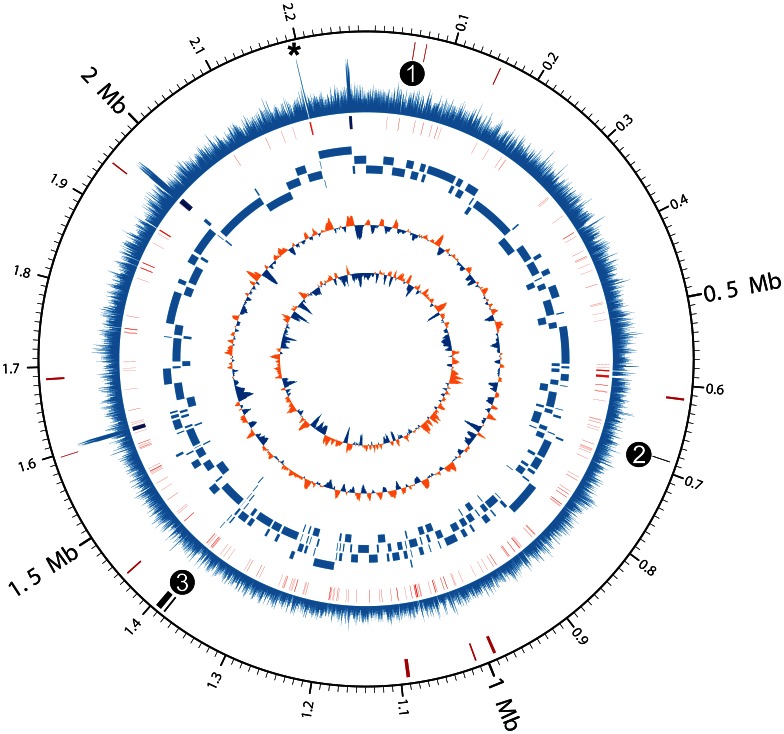
Circular representation of *R. mucilaginosa* CF1E draft genome. Genome coordinates are given in Mbp. From outside to inside, the circles represent: (i) Fragments missing in the DY-18 reference (red); CRISPR region and phage-associated genes as 1) phage lysin, 2) phage shock protein, and 3) CRISPR elements, (ii) Coverage of the genome up to the scale of 50×; *marked Region V containing a genome fragment with an average coverage of 38×(peaks at 48×) at the region of rearrangement hotspot (*rhs*) elements (iii) Gaps in the scaffold (red); rRNA operons (blue) (iv) Contig order and size; (v) GC skew; (vi) GC content deviation.

The metagenome from CF1E had over 40,000 reads mapping to the reference genome, indicating enough data may be present to reconstruct the full genome of the *R. mucilaginosa* strain present. All CF1E metagenomic reads were assembled *de novo* into 996 contigs with a N50 value (weighted median value of all contigs) of 11,178 bp. Contigs were aligned against the reference genome *R. mucilaginosa* DY-18 using nucmer [44], resulting in one single scaffold built from 181 contigs with an 8.8-fold average sequencing depth (Figure 3). The CF1E *R. mucilaginosa* genome scaffold was then annotated using the RAST server (Genome ID: 43675.9) and compared to DY-18 that had been re-annotated using the same pipeline.

The CF1E genome scaffold consists of one circular chromosome of 2,278,618 bp with a GC content of 59.6%. Only large indels are reported here and SNPs were not examined. No large rearrangements were detected between CF1E and the reference genome DY-18. Phylogenetic analysis of the 16S rDNA loci indicated CF1E and the reference strain DY-18 are close relatives (Supporting Information S3), which is consistent with their average pairwise nucleotide identity of 85%. The sequence reads were relatively equally distributed across the genome, except at the multi-copy rRNA genes and in the highly conserved *rhs* region (Figure 3).

### The High Coverage Regions – rRNA Operons and rhs Elements

The rRNA operons assembled into one single contig (contig173) and had an average coverage depth of approximately 3.5 times the average depth of coverage for the rest of the scaffold (i.e. 31X versus 8.8X). The sequence from this contig was used to fill in three gaps that were predicted to correspond to the rRNA operons, based on alignment with the reference genome (Figure 3).

The rearrangement hot spot (*rhs*) gene region (Figure 3: Region V marked *) also had a high average coverage of 39X. The primary structure of Rhs proteins consists of an N-terminal domain, a “core” domain, a hyperconserved domain, and a DPxGL motif followed by a C-terminus that varies between strains and species [45]. Previous studies have shown that *rhs* genes play a role in competition between strains or species, similar to the contact-dependent growth inhibition (CDI) system [46]. The variable C-termini of Rhs proteins have toxin activities, and the small genes that typically follow *rhs* genes are thought to encode proteins that provide immunity to the toxins. Kung *et al.* (2012) showed that the *rhs-CT* in *P. aeruginosa* delivers toxins to eukaryotic cells, activating the inflammasome [47]. The high coverage of the conserved *rhs* region suggests that *rhs* is present in high abundance in the CF microbial community. It is possible that the *rhs* system is widely used by CF microbes for (i) cell-to-cell interactions and communication, particularly for biofilm formation, (ii) direct antagonistic effects on the growth or viability of competitors, and/or (iii) attacking cells of the host-immune system. Additional experimental studies are needed to further assess these possibilities.

The high coverage *rhs* region in the CF1E genome scaffold included an *rhs* gene sequence related to one of the two *rhs* genes of DY-18 (RMDY18_19250). However, there is an apparent gap in the scaffold sequence, beginning 24 amino acids upstream of the DPxGL motif of the encoded Rhs protein. In the DY-18 reference genome, this gap corresponds to the coding sequence for the toxic C-terminal region of Rhs, and the beginning of the gene encoding the RhsI immunity protein. Assuming the presence of multiple *rhs-CT/rhsI* modules in the metagenome, assembling this region will be challenging.

### Functional Annotation of the *R. mucilaginosa* Genome Scaffold

RAST predicted 1,739 gene products belonging to 248 function subsystems (Table S5). The most abundant functions included biosynthesis and degradation of amino acids and derivatives, protein metabolism, cofactor/vitamin/prosthetic group/pigment biosynthesis and metabolism, and carbohydrate metabolism (Table S6). Thirty-seven ORFs present in the DY-18 genome and absent in the CF1E scaffold are listed in Table 1 (DY-18 specific). Genes only present in CF1E are listed in Table 2 (CF1E specific). Genome regions specific to only one of the two strains ranged from multiple kbp (mostly in gene coding regions) to a few nucleotides in non-coding regions. Additional analyses were performed on several of the genomic regions unique to CF1E; regions were chosen for their potential influences on CF-lung specific evolution of niche utilization and antibiotic resistance.

**Table 1:** Genomic regions present in the DY-18 reference genome but missing from the CF1E draft genome.

Region starting coordinate(on DY-18)	Region size (bp)	Protein(s) annotated on the genomic fragment
44,199	697	Predicted ARSR subfamily of helix-turn-helix bacterial transcription regulatory protein CDS
119,455	7,752	Conserved hypothetical protein, putative cell filamentation protein CDS
138,835	470	Predicted nucleic acid-binding protein CDS; exopolysaccharide biosynthesis protein related to N-acetylglucosamine-1-phosphodiester alpha-N-acetylglucosaminidase CDS
149,644	1,290	UBA/THIF-type NAD/FAD binding fold CDS
319,311	5,105	ATP-binding protein of ABC transporter
620,953	3,564	Putative glutamate transporter permease protein CDS; ABC-type amino acid transport system, permease component CDS; Glutamate binding protein CDS; COG1126: ABC-type polar amino acid transport system, ATPase component CDS
1,410,305	915	Putative integral membrane protein CDS
1,817,923	2,084	Pyruvate oxidase [ubiquinone, cytochrome] (EC 1.2.2.2) CDS
1,924,698	994	Cell wall surface anchor family protein CDS
2,084,618	5,926	Amino acid ABC transporter, periplasmic amino acid-binding protein CDS; Cystathionine gamma-lyase (EC 4.4.1.1) CDS; O-acetylhomoserine sulfhydrylase (EC 2.5.1.49) CDS
Total of 20		Hypothetical proteins*

See details in Table S11.

**Table 2:** Predicted protein-coding sequences present in the CF1E scaffold annotated by RAST, but missing from the DY-18 reference (list excludes hypothetical proteins).

Inserted position(based on DY-18)	Fragment length (bp)	Protein annotated on the inserted fragment
53,748	3,174	Phage lysin, N-acetylmuramoyl-L-alanine amidase CDS
65,967	913	Modulator of drug activity B
70,542	1,534	Putative DNA-binding protein
156,926	1,615	Predicted nucleic acid-binding protein CDS
239,949	1,690	Putative hydrolase CDS
613,830	2,685	Acyltransferase 3 CDS
808,821	318	Mobile element protein CDS
1,013,020	1,866	2-oxoglutarate/malate translocator CDS
1,671,769	3,475	Macrolide export ATP-binding/permease protein MacB (EC 3.6.3.-) CDS
1,884,458	1,840	Mobile element protein CDS
2,044,067	1,547	L-lactate dehydrogenase (EC 1.1.2.3) CDS
Total of 21		Hypothetical proteins*

See details in Table S12.

- *L-lactate dehydrogenases (LDHs):* The CF1E scaffold had a cytochrome c-dependent LDH (EC 1.1.2.3) in addition to the expected NAD (P)-dependent LDH (EC 1.1.1.27). The nucleotide sequence of LDH (EC 1.1.2.3) was 80% identical to the LDH of *R. dentocariosa* ATCC 17931. Lactate is secreted by the human host, and produced by many CF-associated microbes (e.g., *Staphylococcus* and *Streptococcus* spp.) through fermentation [48]. Lactate has been detected in the CF sputum at a mean concentration of 3 mM, and higher concentrations have been correlated with lower lung function [49]. Because LDHs enable cells to use lactate as an energy source for growth and reproduction, they are considered as virulence factors. For example, utilization of lactate by *Neisseria* spp. (reviewed in [50]) enhances their rate of O_2_ metabolism [51].
*R. mucilaginosa* is a facultative anaerobe. The presence of both types of LDH may allow cells to respond to micro-changes in oxygen and nutrient availability by utilizing different metabolic pathways. This would indicate that the primary niche of *R. mucilaginosa* is the microaerophilic environment at the epithelial surfaces in the mucus plug, which also contains lactate and oxygen from the cells and blood, respectively. A cytochrome c-dependent LDH could allow *R. mucilaginosa* to utilize extracellular L-lactate with cytochrome c as the terminal oxidase [52] under aerobic conditions, producing pyruvate and hydrogen peroxide (H_2_O_2_) [53]. Pyruvate could serve as a food reservoir for fermentative bacteria (e.g., *R. mucilaginosa* in the CF lung [54]) while also inhibiting the glucose uptake rate of competing bacteria [55]. The production of H_2_O_2_ could also serve to inhibit the growth of other organisms, or be used by microbes with catalase activity to yield water that is scarce in the dehydrated CF lung environment [56], [57]. Under anaerobic conditions, NAD-dependent LDH allows the organism to undergo fermentation through the reduction of pyruvate to lactate (reviewed in Garvie E.I. [53]).
- *Antibiotic resistant genes:* An additional copy of a gene encoding the macrolide export ATP-binding/permease protein MacB was found into the CF1E scaffold. Sequence alignments showed that the two MacB-encoding genes are only 12% identical at the nucleotide level, indicating that one MacB was acquired horizontally and did not originate by gene duplication. The protein sequence of the acquired MacB matched a hypothetical protein in *R. mucilaginosa* M508 and MacB from *R. mucilaginosa* ATCC 25296 (E-value: 0). The predicted amino acid sequences showed specific hits to the family comprising the MJ0796 ATP-binding cassette (CD03255), followed by a MacB-like periplasmic core domain (PFAM 12704) and FtsX-like permease family (PFAM02687) domain.
  In addition, modulator of drug activity B (MdaB) was present in the CF1E scaffold. Overexpression of MdaB has been shown to confer resistance against tetracycline and adriamycin in *E. coli* [58]. In addition to this gene, the genome of *R. mucilaginosa* in CF1E encoded drug resistance transporters (EmrB/QacA subfamily), multidrug resistance transporters (Bcr/CflA family), and a glycopeptide antibiotic resistance protein. These diverse strategies for antibiotic resistance may underlie *R. mucilaginosa’s* ability to survive antibiotic treatments (Figure 1).
- *Type I restriction modification:* The type I restriction modification (R-M) system is a mechanism to protect against foreign nucleic acids via non site specific endonucleases [59]. There are three subunits: M (Modification/Methyltransferase), S (Specificity) and R (Restriction). The M and S subunits are responsible for recognizing self and non-self, while the R subunit performs the cleavage. The S subunit contains two target recognition domains that are important for restriction specificity and modification of the complex activity. Mapping of metagenomic reads to the reference DY-18 genome (Figure 2, Table S3) showed that only the CF1E metagenome had this Type I R-M system region, whereas the other metagenomes had gaps of 7–9 kbp around this region of the DY-18 genome. The CF1E scaffold likely encodes an S subunit with different sequence specificity, as this subunit is only 37% identical (nucleotides) or 41% identical (protein) to the DY-18 copy (Table S7 and Figure S1). This is of interest because Type I R-M systems have been modified during the adaptation of *P. aeruginosa* and *Burkholderia cenocepacia* to the CF lung. For example, the Type I R-M of *P. aeruginosa* Liverpool epidemic strain (LES) colonizing CF patients was shown to carry a different regulatory specificity (M-subunit) in comparison to strain PA01 [60]. In addition, the expression of type I R-M was greatly increased in *B. cenocepacia* in the presence of sub-inhibitory concentrations of antibiotics [61]. Together these observations suggest that modification of type I R-M system could be a general mechanism for adaptation to the CF lung.
- *Phage lysin:* Phage lysins are anti-bacterial agents often used in bacterial competition, and have also been associated with the release of cellular components to the extracellular medium during biofilm formation [62], [63]. One copy of the phage lysin gene was present in CF1E, but this did not have any appreciable nucleotide similarity to any genes in phage or bacteria. However, bioinformatic analysis of the predicted amino acid sequence revealed its similarity to the N-acetylmuramoyl-L-alanine amidase of *R. mucilaginosa* ATCC strain 25296 (E-value: 10^−150^), a hypothetical protein of *R. mucilaginosa* M508 (E-value: 10^−148^), and an amidase-5 domain similar to pneumococcal bacteriophage Dp-1 (E-value: 6.88×10^−42^). Phage lysins are commonly found in prophages [64]. However, no prophages were detected in the CF1E genome scaffold based on PhiSpy [65]. Although it is currently unclear what, if any, advantage is offered by this phage lysin in the *R. mucilaginosa* genome, this lysin could provide an alternative strategy for microbial competition.
- *Clusters of interspaced short palindromic repeats (CRISPRs):* CRISPRs are characterized by stretches of short sequence repeats that flank short “spacer” sequences composed of viral or plasmid DNA. Four CRISPR elements were identified in CF1E; these were all ∼4 kbp downstream of the Cas1 CRISPR-associated gene. The length of these CRISPRs ranged from 253 bp to 1,316 bp (Table S8). All CRISPRs contained the same direct repeat sequence of 36 bp. The spacers in each CRISPR element (collectively referred to as a ‘spacer set’) ranged in copy number from 3 to 17, and their sizes ranged from 33 bp to 88 bp. Two of the spacer sets code for hypothetical proteins while the other two sets are unknown (Table S8). A total of 48 spacer sequences were extracted from the four spacer sets; these spacers were compared to the CF1E virome sequences, but no similarities were found.

Phages are an important source of genes in microbial communities. The CRISPRs found in *R. mucilaginosa* CF1E may correspond to previously attacking phages and plasmids that these cells were able to resist. In order to identify these phage perpetrators, spacer sequences were compared against all host-associated and environmental viromes in MyMgDB [66]. One of the spacers was identified in two human oral cavity viromes [67], whereas none of the spacers were similar to sequences from other environmental viromes (Table S9). The results suggest these bacteria may have been exposed to phages found in the oral cavity, which suggests cells may have existed in this environment prior to opportunistic infection of the CF lungs. Because these spacer sequences did not match phages in the virome sequenced from the same sample, the phages to which *R. mucilaginosa* is resistant are not present, or are below the detection limit, in this sample. However, if temperate phages dominate in the CF lung [67] as in the human gut virome [68], this result is expected because the virome would largely composed of free-living viruses. However it is also possible that these CRISPRs do not protect the cells against phage infection, but are involved in a CRISPR-dependent modulation of biofilm formation, as described previously in *P. aeruginosa* (reviewed in [69]). Biofilm formation has been shown to be important for persistent bacterial infection of CF lungs, as well as an overall decline in lung function. Therefore, the role of these CRISPRs in CF1E and other CF lung isolates’ pathogenesis should be explored further.

### Conclusions

The metagenomic and genomic analyses presented here suggest that *R. mucilaginosa* is a common inhabitant of CF lungs, and that it evolves and adapts to each patient’s lung environment over the course of a persistent infection. Genomic analysis of CF1E highlighted many potential adaptations: multiple genes encoding L-lactate dehydrogenases (LDHs) that could enable utilization of lactate, many multi-drug efflux pumps for antibiotic resistance, and the modification of *rhs* elements and the type I restriction system. Alterations of the type I restriction system has the potential to influence horizontal transfer of genes. The CF1E genomic sequence indicates extensive phage-host interactions, including the acquisition of a phage lysin and changing CRISPR elements.

**Figure 4:**
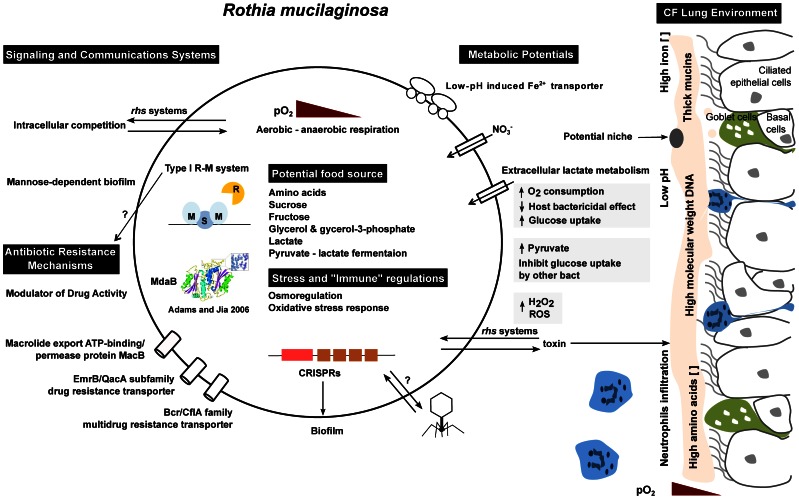
Hypothesized adaptions of *R. mucilaginosa* to the CF lung environment. The model is based on the comparison between the reference genome DY-18 and the reconstructed genome CF1E. *rhs*: rearrangement hot spot; Type I R-M: Type I restriction modification; MdaB: Modular of drug activity B; ROS: reactive oxygen species; CRISPR: Clustered Regularly Interspaced Short Palindromic Repeats.

Based on these potential metabolic adaptations, we hypothesize that *R. mucilaginosa* lives in the microaerophilic surface of the viscous mucus layer that is characteristic of CF airways (Figure 4). Under this hypothesis, cytochrome c-dependent LDH would enable *R. mucilaginosa* to use extracellular lactate. However, this process would require oxygen, which is more readily available at the surface of the mucus layer (e.g., from the blood). As the oxygen level is depleted, metabolism could be supported by fermentation and anaerobic respiration with nitrate as an alternative electron acceptor, as observed in *P. aeruginosa* [70]. Persistence in low oxygen environments would also allow for evasion of antibiotics and ROS activity. In addition, *R. mucilaginosa* carries a low-pH induced ferrous ion (Fe^2+^) transporter along with heme and hemin uptake and utilization systems. Co-occurring CF pathogens including *P. aeruginosa* and *S. maltophilia* are known to synthesize redox active phenazines that are able to reduce Fe^3+^ to Fe^2+^ [71], [72] potentially giving *R. mucilaginosa* access to Fe^2+^ in the low pH sputum where the ferrous ion transporter is induced.

The results presented here highlight the similar evolutionary trajectories and ecological niches of several species of bacteria that colonize the CF lung. These similarities are remarkable because each bacterial species starts with different genetic material: *P. aeruginosa* has a relatively large genome (>6 Mbp), whereas *R. mucilaginosa* has only a 2 Mbp genome (Table S10). These findings suggest that obtaining strain specific genome data can illuminate patient-specific bacterial inhabitants of CF patients. This specific information enables predictions to be made regarding the bacteria’s physiological adaptations in each patient, which would further enable physicians to optimize antibiotic treatments.

## Materials and Methods

### Microbial Metagenome Data

Induced sputum samples were collected from CF volunteers at the Adult CF Clinic (San Diego, CA, United States) by expectoration. All collection was approved by the University of California Institutional Review Board (HRPP 081500) and San Diego State University Institutional Review Board (SDSU IRB#2121). Written informed consent was provided by study participants and/or their legal guardians. Fresh CF sputum samples were processed as described in [12]. In brief, sputum samples were homogenized, bacterial cells were pelleted by centrifugation, and pellets were repeatedly washed and then treated with DNase to remove human DNA prior to extraction of bacterial DNA.

### Sequence Read Processing

A total of 18 microbiomes were previously sequenced using Roche-454 GSFLX [12]. The data were downloaded from NCBI sequence read archive (Accession # SRP009392). Reads that were duplicates or of low quality were removed using PRINSEQ [73], and those that matched human-derived sequences were removed using DeconSeq [74]. Sequence reads with similarity to the phylum Chordata and to vector or synthetic sequences were identified by BLASTn against NCBI nucleotide database (threshold of 40% identity over at least 60% query coverage), and removed from the metagenomes. A detailed description of sample processing and preliminary analyses of these datasets has been published [12].

### BWA Mapping of the Metagenomes

The processed metagenomic reads were mapped to the *Rothia mucilaginosa* DY-18 (GI: 283457089) reference genome using a modified version of BWA-SW 0.5.9. The coverage values based on the reference mapping are shown in Table S4.

### *De novo* Assembly and Scaffolding

The metagenomic reads from CF1E were *de novo* assembled using the Newbler software version 2.6 with ≥35 bp overlap and ≥95% identity. All resultant contigs were aligned to the reference genome (*R. mucilaginosa* DY-18) using nucmer with its -maxmatch option (using all anchor matches regardless of their uniqueness). This option will allow repetitive or multi-copy sequences (e.g., rRNA operons) to assemble into a single contig, enabling that contig to be subsequently mapped to more than one genomic region. All alignments were examined manually. Full length contigs were ordered based on their coordinates on the reference alignment, and this ordering was used with an in-house Perl script to build the final scaffold containing 181 contigs.

### Genome Annotation

The CF1E scaffold was annotated using the RAST web annotation service [75] with the latest FIGfams version 57 (Genome ID: 43675.9). In order to allow a direct comparison, the reference genomes of *R. mucilaginosa*, DY-18 and *R. mucilaginosa* M508 (downloaded from the Genome OnLine Database) were also annotated using the same pipeline. CRISPR loci were identified using CRISPRFinder [76]. The spacers between the repeats were extracted and compared to the virome sequenced from the same sample (downloaded from the NCBI (SRX090639)) [12], and other viromes in mymgdb [66].

### Rothia-targeted 16S PCR of Lung Sections from Explanted Lungs

DNA was extracted from 5–6 homogenized lung tissues from explanted lungs of four transplant patients using the Macherey-Nagel Nucleospin Tissue Kit (Macherey-Nagel, Bethlehem, PA) with the Gram-positive variation that included an overnight proteinase K digestion. Extracted DNA was amplified using Actinobacteria-targeted PCR primers (Rothia_1F: 5′-GGGACATTCCACGTTTTCCG-3′, Rothia_1R: 5′-TCCTATGAGTCCCCACCATT-3′) that encompass a 322 bp region of the 16S rRNA gene including the hypervariable regions 6–7. Two of the four patients were positive for Actinobacteria; right lower and lingular (left) lobes for Lung 9, and lingular lobe for Lung 7 (Supporting Information S3). The PCR products were purified and sequenced. Sequencing of the three partial 16S gene fragments indicated *Rothia* was present in lungs from one of the four CF patients.

## Supporting Information

Figure S1 **Dot Plot matrix view of the alignment of CF1E type I restriction modification system (subunit M, R, S) against DY-18.**
(PDF)

Table S1 **Diseases associated with** ***R. mucilaginosa.***
(PDF)

Table S2 **Microbiomes used in this study.** Clinical status was designated as *exacerbation* (prior to systemic antibiotic treatment), *on treatment* (during systemic antibiotic treatment), *post treatment* (upon completion of systemic antibiotic treatment) or *stable* (when clinically stable and at their clinical and physiological baseline). The samples collected during exacerbation were designated as Day 0 sample, and the times between samples are cumulatively calculated from Day 0.
(PDF)

Table S3 (A) Annotation of the gaps ≥5 kbp in CF1 metagenomic reference mapping against *R. mucilaginosa* DY-18. Refer to Table S2 for detailed patient samples information. (**B)** Annotation of the gaps ≥5 kbp in CF6 metagenomic reference mapping against *R. mucilaginosa* DY-18. Refer to Table S2 for detailed patient samples information. (**C)** Annotation of the gaps ≥5 kbp in CF7 metagenomic reference mapping against *R. mucilaginosa* DY-18. Refer to Table S2 for detailed patient samples information. (**D)** Annotation of the gaps ≥5 kbp in CF8 metagenomic reference mapping against *R. mucilaginosa* DY-18. Refer to Table S2 for detailed patient samples information.
(PDF)

Table S4 **Statistics from BWA mapping of metagenomic reads against the reference genome** ***R. mucilaginosa*** **DY-18.**
(PDF)

Table S5 **General features of the CF1E** ***R. mucilaginosa*** **scaffold, DY-18 reference genome, and M508 draft genome.**
(PDF)

Table S6 **Subsystem feature counts of** ***R. mucilaginosa*** **CF1E, DY-18, and M508.**
(PDF)

Table S7 **Sequence identities of the genes encoding the Type I restriction modification system in CF1E and DY-18, determined using BLAST.** The identity value is subjected to >97% query length coverage.
(PDF)

Table S8 **CRISPR positions in the CF1E genome scaffold.**
(PDF)

Table S9 **Identification of the spacer sequences in CF1E CRISPR structure from human- and environmental-viral metagenomes at 100% length coverage and ≥90% identity (≤2 mismatches).**
(PDF)

Table S10 **A comparison of putative adaptations and predicted metabolisms of** ***R. mucilaginosa*** **and** ***P. aeruginosa*** **that are hypothesized to enable persistence in the CF lung, based on literature and genomic data.**
(PDF)

Table S11 **Genes that are missing from the CF1E genome scaffold but present in the DY-18 reference.** Genes are considered missing when the gap is within a contig.
(PDF)

Table S12 **Genes present in the CF1E genome scaffold but missing in the reference genome DY-18.**
(PDF)

Table S13 **Isolation source and references of sequences extracted and used in the 16S phylogenetic analysis.**
(PDF)

Table S14 **Protein-coding genes used for multilocus phylogenetic inference.**
(PDF)

Table S15 **Genes missing from the CF1E genome scaffold, based on contig mapping to the reference genome DY18.**
(PDF)

Supporting Information S1 **Additional samples information.**
(DOCX)

Supporting Information S2 ***Rothia mucilaginosa*** **in cystic fibrosis community metatranscriptomes.**
(DOCX)

Supporting Information S3 **Additional genome characteristic of** ***R. mucilaginosa*** **and phylogenetic analysis of** ***Rothia*** **spp. associated with cystic fibrosis.**
(DOCX)

## References

[1] Kerem B, Rommens JM, Buchanan JA, Markiewicz D, Cox TK, et al (1989) Identification of the Cystic Fibrosis gene: Genetic analysis. Science 245: 1073–1080 doi:10.1126/science.2570460 2570460 10.1126/science.2570460

[2] LiPuma JJ (2010) The changing microbial epidemiology in Cystic Fibrosis. Clinical Microbiology Reviews 23: 299–323 doi:10.1128/CMR.00068-09 20375354 10.1128/CMR.00068-09 PMC2863368

[3] Rogers GB, Carroll MP, Serisier DJ, Hockey PM, Jones G, et al (2004) Characterization of bacterial community diversity in Cystic Fibrosis lung infections by use of 16S ribosomal DNA terminal restriction fragment length polymorphism profiling. J Clin Microbiol 42: 5176–5183 doi:10.1128/JCM.42.11.5176-5183.2004 15528712 10.1128/JCM.42.11.5176-5183.2004 PMC525137

[4] Harris JK, De Groote MA, Sagel SD, Zemanick ET, Kapsner R, et al (2007) Molecular identification of bacteria in bronchoalveolar lavage fluid from children with Cystic Fibrosis. PNAS 104: 20529–20533 doi:10.1073/pnas.0709804104 18077362 10.1073/pnas.0709804104 PMC2154465

[5] Bittar F, Richet H, Dubus J-C, Reynaud-Gaubert M, Stremler N, et al.. (2008) Molecular detection of multiple emerging pathogens in sputa from Cystic Fibrosis patients. PLoS ONE. doi:10.1371/journal.pone.0002908. 10.1371/journal.pone.0002908 PMC2483419 18682840

[6] Cox MJ, Allgaier M, Taylor B, Baek MS, Huang YJ, et al (2010) Airway microbiota and pathogen abundance in age-stratified Cystic Fibrosis patients. PLoS ONE 5: e11044 doi:10.1371/journal.pone.0011044 20585638 10.1371/journal.pone.0011044 PMC2890402

[7] Guss AM, Roeselers G, Newton ILG, Young CR, Klepac-Ceraj V, et al (2011) Phylogenetic and metabolic diversity of bacteria associated with cystic fibrosis. ISME J 5: 20–29. 20631810 10.1038/ismej.2010.88 PMC3105664

[8] Willner D, Furlan M, Haynes M, Schmieder R, Angly FE, et al (2009) Metagenomic analysis of respiratory tract DNA viral communities in Cystic Fibrosis and non-Cystic Fibrosis individuals. PLoS ONE 4: e7370 doi:10.1371/journal.pone.0007370 19816605 10.1371/journal.pone.0007370 PMC2756586

[9] Willner D, Furlan M (2010) Deciphering the role of phage in the cystic fibrosis airway. Virulence 1: 309–313 doi:10.4161/viru.1.4.12071 21178461 10.4161/viru.1.4.12071

[10] Willner D, Haynes MR, Furlan M, Schmieder R, Lim YW, et al (2011) Spatial distribution of microbial communities in the cystic fibrosis lung. The ISME Journal 6: 471–474 doi:10.1038/ismej.2011.104 21796216 10.1038/ismej.2011.104 PMC3260497

[11] Willner D, Haynes MR, Furlan M, Hanson N, Kirby B, et al (2012) Case studies of the spatial heterogeneity of DNA viruses in the cystic fibrosis lung. Am J Respir Cell Mol Biol 46: 127–131 doi:10.1165/rcmb.2011-0253OC 21980056 10.1165/rcmb.2011-0253OC PMC3361360

[12] Lim YW, Schmieder R, Haynes M, Willner D, Furlan M, et al.. (2012) Metagenomics and metatranscriptomics: Windows on CF-associated viral and microbial communities. J Cyst Fibros. doi:10.1016/j.jcf.2012.07.009. 10.1016/j.jcf.2012.07.009 PMC3534838 22951208

[13] Narasingarao P, Podell S, Ugalde JA, Brochier-Armanet C, Emerson JB, et al (2012) De novo metagenomic assembly reveals abundant novel major lineage of Archaea in hypersaline microbial communities. ISME J 6: 81–93 doi:10.1038/ismej.2011.78 21716304 10.1038/ismej.2011.78 PMC3246234

[14] Iverson V, Morris RM, Frazar CD, Berthiaume CT, Morales RL, et al (2012) Untangling genomes from metagenomes: revealing an uncultured class of marine Euryarchaeota. Science 335: 587–590 doi:10.1126/science.1212665 22301318 10.1126/science.1212665

[15] Migula W (1900) System der bakterien: bd. Specielle systematik der bakterien. G. Fischer. 1164 p.

[16] Bergan T, Bøvre K, Hovig B (1970) Priority of Micrococcus mucilaginosus Migula 1900 over Staphylococcus salivarius: Andrewes and Gordon 1907 with proposal of a neotype strain. International Journal of Systematic Bacteriology 20: 107–113 doi:10.1099/00207713-20-1-107

[17] Bergan T, Kocur M (1982) NOTES: Stomatococcus mucilaginosus gen.nov., sp.nov., ep. rev., a member of the family Micrococcaceae. International Journal of Systematic Bacteriology 32: 374–377 doi:10.1099/00207713-32-3-374

[18] Collins MD, Hutson RA, Båverud V, Falsen E (2000) Characterization of a Rothia-like organism from a mouse: description of Rothia nasimurium sp. nov. and reclassification of Stomatococcus mucilaginosus as Rothia mucilaginosa comb. nov. International Journal of Systematic and Evolutionary Microbiology 50: 1247–1251. 10843069 10.1099/00207713-50-3-1247

[19] Doel JJ, Benjamin N, Hector MP, Rogers M, Allaker RP (2005) Evaluation of bacterial nitrate reduction in the human oral cavity. European Journal of Oral Sciences 113: 14–19 doi:10.1111/j.1600-0722.2004.00184.x 15693824 10.1111/j.1600-0722.2004.00184.x

[20] Olsen I, Preza D, Aas JA, Paster BJ (2009) Cultivated and not-yet-cultivated bacteria in oral biofilms. Microbial Ecology in Health and Disease 21: 65–71 doi:10.1080/08910600902907509

[21] Guglielmetti S, Taverniti V, Minuzzo M, Arioli S, Stuknyte M, et al (2010) Oral bacteria as potential probiotics for the pharyngeal mucosa. Applied and Environmental Microbiology 76: 3948–3958 doi:10.1128/AEM.00109-10 20418429 10.1128/AEM.00109-10 PMC2893495

[22] Wang M, Ahrné S, Jeppsson B, Molin G (2005) Comparison of bacterial diversity along the human intestinal tract by direct cloning and sequencing of 16S rRNA genes. FEMS Microbiology Ecology 54: 219–231 doi:10.1016/j.femsec.2005.03.012 16332321 10.1016/j.femsec.2005.03.012

[23] Ou G, Hedberg M, Horstedt P, Baranov V, Forsberg G, et al. (2009) Proximal small intestinal microbiota and identification of rod-shaped bacteria associated with childhood celiac disease. Am J Gastroenterol. Available: http://dx.doi.org/10.1038/ajg.2009.524. Accessed 2012 Feb 20. 10.1038/ajg.2009.524 19755974

[24] Kazor CE, Mitchell PM, Lee AM, Stokes LN, Loesche WJ, et al (2003) Diversity of bacterial populations on the tongue dorsa of patients with halitosis and healthy patients. Journal of Clinical Microbiology 41: 558–563 doi:10.1128/JCM.41.2.558-563.2003 12574246 10.1128/JCM.41.2.558-563.2003 PMC149706

[25] Preza D, Olsen I, Willumsen T, Grinde B, Paster BJ (2009) Diversity and site-specificity of the oral microflora in the elderly. European Journal of Clinical Microbiology & Infectious Diseases 28: 1033–1040 doi:10.1007/s10096-009-0743-3 19373498 10.1007/s10096-009-0743-3 PMC2821189

[26] Nyvad B, Kilian M (1987) Microbiology of the early colonization of human enamel and root surfaces in vivo. European Journal of Oral Sciences 95: 369–380 doi:10.1111/j.1600-0722.1987.tb01627.x 10.1111/j.1600-0722.1987.tb01627.x 3477852

[27] Philip K, Teoh WY, Muniandy S, Yaakob H (2009) Pathogenic bacteria predominate in the oral cavity of Malaysian subjects. Available: http://www.scialert.net/pdfs/jbs/2009/438-444.pdf, http://www.doaj.org/doaj?func=openurl&genre=article&issn=17273048&date=2009&volume=9&issue=5&spage=438. Accessed 2012 Feb 22.

[28] Jiménez E, Delgado S, Fernández L, García N, Albújar M, et al (2008) Assessment of the bacterial diversity of human colostrum and screening of staphylococcal and enterococcal populations for potential virulence factors. Research in Microbiology 159: 595–601 doi:10.1016/j.resmic.2008.09.001 18845249 10.1016/j.resmic.2008.09.001

[29] Delgado S, Arroyo R, Martín R, Rodríguez JM (2008) PCR-DGGE assessment of the bacterial diversity of breast milk in women with lactational infectious mastitis. BMC Infectious Diseases 8: 51 doi:10.1186/1471-2334-8-51 18423017 10.1186/1471-2334-8-51 PMC2383900

[30] Bowden GH (1969) The components of the cell walls and extracellular slime of four strains of Staphylococcus salivarius isolated from human dental plaque. Archives of Oral Biology 14: 685–697 doi:10.1016/0003-9969(69)90190-3 5257939 10.1016/0003-9969(69)90190-3

[31] Ready D, Lancaster H, Qureshi F, Bedi R, Mullany P, et al (2004) Effect of amoxicillin use on oral microbiota in young children. Antimicrobial Agents and Chemotherapy 48: 2883–2887 doi:10.1128/AAC.48.8.2883-2887.2004 15273096 10.1128/AAC.48.8.2883-2887.2004 PMC478491

[32] Stackebrandt E (2006) The Genus Stomatococcus: Rothia mucilaginosa, basonym Stomatococcus mucilaginosus. In: Dworkin M, Falkow S, Rosenberg E, Schleifer K-H, Stackebrandt E, editors. The Prokaryotes. Springer New York, Vol. 3. 975–982. Available: http://www.springerlink.com/content/v05011710446lr65/. Accessed 2012 Feb 19.

[33] Tunney MM, Field TR, Moriarty TF, Patrick S, Doering G, et al (2008) Detection of anaerobic bacteria in high numbers in sputum from patients with cystic fibrosis. Am J Respir Crit Care Med 177: 995–1001 doi:10.1164/rccm.200708-1151OC 18263800 10.1164/rccm.200708-1151OC

[34] Van der Gast CJ, Walker AW, Stressmann FA, Rogers GB, Scott P, et al (2011) Partitioning core and satellite taxa from within Cystic Fibrosis lung bacterial communities. ISME J 5: 780–791. 21151003 10.1038/ismej.2010.175 PMC3105771

[35] Rogers GB, Carroll MP, Serisier DJ, Hockey PM, Jones G, et al (2006) Use of 16S rRNA gene profiling by terminal restriction fragment length polymorphism analysis to compare bacterial communities in sputum and mouthwash samples from patients with Cystic Fibrosis. J Clin Microbiol 44: 2601–2604 doi:10.1128/JCM.02282-05 16825392 10.1128/JCM.02282-05 PMC1489498

[36] Goddard AF, Staudinger BJ, Dowd SE, Joshi-Datar A, Wolcott RD, et al (2012) Direct sampling of cystic fibrosis lungs indicates that DNA-based analyses of upper-airway specimens can misrepresent lung microbiota. PNAS 109: 13769–13774 doi:10.1073/pnas.1107435109 22872870 10.1073/pnas.1107435109 PMC3427132

[37] Fodor AA, Klem ER, Gilpin DF, Elborn JS, Boucher RC, et al (2012) The adult cystic fibrosis airway microbiota is stable over time and infection type, and highly resilient to antibiotic treatment of exacerbations. PLoS ONE 7: e45001 doi:10.1371/journal.pone.0045001 23049765 10.1371/journal.pone.0045001 PMC3458854

[38] Gomes-Filho IS, Passos JS, Seixas da Cruz S (2010) Respiratory disease and the role of oral bacteria. J Oral Microbiol 2. doi:10.3402/jom.v2i0.5811. 10.3402/jom.v2i0.5811 PMC3084574 21523216

[39] Oliver A, Cantón R, Campo P, Baquero F, Blázquez J (2000) High frequency of hypermutable Pseudomonas aeruginosa in cystic fibrosis lung infection. Science 288: 1251–1253 doi:10.1126/science.288.5469.1251 10818002 10.1126/science.288.5469.1251

[40] Lieberman TD, Michel J-B, Aingaran M, Potter-Bynoe G, Roux D, et al (2011) Parallel bacterial evolution within multiple patients identifies candidate pathogenicity genes. Nature Genetics 43: 1275–1280 doi:10.1038/ng.997 22081229 10.1038/ng.997 PMC3245322

[41] Goerke C, Wolz C (2010) Adaptation of Staphylococcus aureus to the cystic fibrosis lung. Int J Med Microbiol 300: 520–525 doi:10.1016/j.ijmm.2010.08.003 20843740 10.1016/j.ijmm.2010.08.003

[42] Yamane K, Nambu T, Yamanaka T, Mashimo C, Sugimori C, et al.. (2010) Complete genome sequence of Rothia mucilaginosa DY-18: A clinical isolate with dense meshwork-like structures from a persistent apical periodontitis lesion. Sequencing 2010. doi:10.1155/2010/457236.

[43] Lawrence JG, Ochman H (1998) Molecular archaeology of the Escherichia coli genome. PNAS 95: 9413–9417. 9689094 10.1073/pnas.95.16.9413 PMC21352

[44] Kurtz S, Phillippy A, Delcher AL, Smoot M, Shumway M, et al (2004) Versatile and open software for comparing large genomes. Genome Biol 5: R12 doi:10.1186/gb-2004-5-2-r12 14759262 10.1186/gb-2004-5-2-r12 PMC395750

[45] Jackson AP, Thomas GH, Parkhill J, Thomson NR (2009) Evolutionary diversification of an ancient gene family (rhs) through C-terminal displacement. BMC Genomics 10: 584 doi:10.1186/1471-2164-10-584 19968874 10.1186/1471-2164-10-584 PMC2935791

[46] Poole SJ, Diner EJ, Aoki SK, Braaten BA, T’ Kint de Roodenbeke C, et al (2011) Identification of functional toxin/immunity genes linked to Contact-Dependent Growth Inhibition (CDI) and Rearrangement Hotspot (Rhs) systems. PLoS Genet 7: e1002217 doi:10.1371/journal.pgen.1002217 21829394 10.1371/journal.pgen.1002217 PMC3150448

[47] Kung VL, Khare S, Stehlik C, Bacon EM, Hughes AJ, et al (2012) An rhs gene of Pseudomonas aeruginosa encodes a virulence protein that activates the inflammasome. PNAS 109: 1275–1280 doi:10.1073/pnas.1109285109 22232685 10.1073/pnas.1109285109 PMC3268321

[48] De Backer D, Creteur J, Zhang H, Norrenberg M, Vincent J-L (1997) Lactate production by the lungs in acute lung injury. Am J Respir Crit Care Med 156: 1099–1104. 9351608 10.1164/ajrccm.156.4.9701048

[49] Bensel T, Stotz M, Borneff-Lipp M, Wollschläger B, Wienke A, et al (2011) Lactate in cystic fibrosis sputum. Journal of Cystic Fibrosis 10: 37–44 doi:10.1016/j.jcf.2010.09.004 20947455 10.1016/j.jcf.2010.09.004

[50] Smith H, Tang CM, Exley RM (2007) Effect of host lactate on gonococci and meningococci: New concepts on the role of metabolites in pathogenicity. Infect Immun 75: 4190–4198 doi:10.1128/IAI.00117-07 17562766 10.1128/IAI.00117-07 PMC1951187

[51] Britigan BE, Klapper D, Svendsen T, Cohen MS (1988) Phagocyte-derived lactate stimulates oxygen consumption by Neisseria gonorrhoeae. An unrecognized aspect of the oxygen metabolism of phagocytosis. J Clin Invest 81: 318–324. 3123517 10.1172/JCI113323 PMC329573

[52] Lederer F (1974) On the first steps of lactate oxidation by bakers’ yeast L-(plus)-lactate dehydrogenase (Cytochrome b2). European Journal of Biochemistry 46: 393–399 doi:10.1111/j.1432-1033.1974.tb03632.x 4152980 10.1111/j.1432-1033.1974.tb03632.x

[53] Garvie EI (1980) Bacterial lactate dehydrogenases. Microbiol Rev 44: 106–139. 6997721 10.1128/mr.44.1.106-139.1980 PMC373236

[54] Price-Whelan A, Dietrich LEP, Newman DK (2007) Pyocyanin alters redox homeostasis and carbon flux through central metabolic pathways in Pseudomonas aeruginosa PA14. J Bacteriol 189: 6372–6381 doi:10.1128/JB.00505-07 17526704 10.1128/JB.00505-07 PMC1951912

[55] Brown SA, Whiteley M (2007) A novel exclusion mechanism for carbon resource partitioning in Aggregatibacter actinomycetemcomitans. J Bacteriol 189: 6407–6414 doi:10.1128/JB.00554-07 17586632 10.1128/JB.00554-07 PMC1951915

[56] Potter JL, Matthews LW, Spector S, Lemm J (1967) Studies on pulmonary secretions. II. Osmolality and the ionic environment of pulmonary secretions from patients with cystic fibrosis, bronchiectasis, and laryngectomy. Am Rev Respir Dis 96: 83–87. 6027728 10.1164/arrd.1967.96.1.83

[57] Tarran R, Grubb BR, Gatzy JT, Davis CW, Boucher RC (2001) The relative roles of passive surface forces and active ion transport in the modulation of airway surface liquid volume and composition. J Gen Physiol 118: 223–236 doi:10.1085/jgp.118.2.223 11479349 10.1085/jgp.118.2.223 PMC2233832

[58] Adams MA, Jia Z (2006) Modulator of drug activity B from Escherichia coli: crystal structure of a prokaryotic homologue of DT-diaphorase. J Mol Biol 359: 455–465 doi:10.1016/j.jmb.2006.03.053 16630630 10.1016/j.jmb.2006.03.053

[59] Murray NE (2000) Type I restriction systems: Sophisticated molecular machines (a legacy of Bertani and Weigle). Microbiol Mol Biol Rev 64: 412–434. 10839821 10.1128/mmbr.64.2.412-434.2000 PMC98998

[60] Smart CHM, Walshaw MJ, Hart CA, Winstanley C (2006) Use of suppression subtractive hybridization to examine the accessory genome of the Liverpool cystic fibrosis epidemic strain of Pseudomonas aeruginosa. J Med Microbiol 55: 677–688 doi:10.1099/jmm.0.46461-0 16687584 10.1099/jmm.0.46461-0

[61] Sass A, Marchbank A, Tullis E, LiPuma JJ, Mahenthiralingam E (2011) Spontaneous and evolutionary changes in the antibiotic resistance of Burkholderia cenocepacia observed by global gene expression analysis. BMC Genomics 12: 373 doi:10.1186/1471-2164-12-373 21781329 10.1186/1471-2164-12-373 PMC3155924

[62] Whitchurch CB, Tolker-Nielsen T, Ragas PC, Mattick JS (2002) Extracellular DNA required for bacterial biofilm formation. Science 295: 1487 doi:10.1126/science.295.5559.1487 11859186 10.1126/science.295.5559.1487

[63] Carrolo M, Frias MJ, Pinto FR, Melo-Cristino J, Ramirez M (2010) Prophage spontaneous activation promotes DNA release enhancing biofilm formation in Streptococcus pneumoniae. PLoS ONE 5: e15678 doi:10.1371/journal.pone.0015678 21187931 10.1371/journal.pone.0015678 PMC3004956

[64] Schmitz JE, Schuch R, Fischetti VA (2010) Identifying active phage lysins through functional viral metagenomics. Appl Environ Microbiol 76: 7181–7187 doi:10.1128/AEM.00732-10 20851985 10.1128/AEM.00732-10 PMC2976241

[65] Akhter S, Aziz RK, Edwards RA (2012) PhiSpy: A novel algorithm for finding prophages in bacterial genomes that combines similarity-based and composition-based strategies. NAR. 10.1093/nar/gks406 PMC3439882 22584627

[66] Schmieder R, Edwards RA (n.d.) MyMGDB. Available: http://edwards.sdsu.edu/cgi-bin/mymgdb.

[67] Willner D, Furlan M, Schmieder R, Grasis JA, Pride DT, et al (2011) Metagenomic detection of phage-encoded platelet-binding factors in the human oral cavity. Proc Natl Acad Sci U S A 108: 4547–4553 doi:10.1073/pnas.1000089107 20547834 10.1073/pnas.1000089107 PMC3063595

[68] Reyes A, Haynes M, Hanson N, Angly FE, Heath AC, et al (2010) Viruses in the faecal microbiota of monozygotic twins and their mothers. Nature 466: 334–338 doi:10.1038/nature09199 20631792 10.1038/nature09199 PMC2919852

[69] Palmer KL, Whiteley M (2011) DMS3–42: the secret to CRISPR-dependent biofilm inhibition in Pseudomonas aeruginosa. J Bacteriol 193: 3431–3432 doi:10.1128/JB.05066-11 21551309 10.1128/JB.05066-11 PMC3133327

[70] Hoffman LR, Richardson AR, Houston LS, Kulasekara HD, Martens-Habbena W, et al (2010) Nutrient availability as a mechanism for selection of antibiotic tolerant Pseudomonas aeruginosa within the CF airway. PLoS Pathog 6: e1000712 doi:10.1371/journal.ppat.1000712 20072604 10.1371/journal.ppat.1000712 PMC2795201

[71] Dietrich LEP, Teal TK, Price-Whelan A, Newman DK (2008) Redox-active antibiotics control gene expression and community behavior in divergent bacteria. Science 321: 1203–1206 doi:10.1126/science.1160619 18755976 10.1126/science.1160619 PMC2745639

[72] Wang Y, Wilks JC, Danhorn T, Ramos I, Croal L, et al (2011) Phenazine-1-carboxylic acid promotes bacterial biofilm development via ferrous iron acquisition. J Bacteriol 193: 3606–3617 doi:10.1128/JB.00396-11 21602354 10.1128/JB.00396-11 PMC3133341

[73] Schmieder R, Edwards R (2011) Quality control and preprocessing of metagenomic datasets. Bioinformatics 27: 863–864 doi:10.1093/bioinformatics/btr026 21278185 10.1093/bioinformatics/btr026 PMC3051327

[74] Schmieder R, Edwards R (2011) Fast identification and removal of sequence contamination from genomic and metagenomic datasets. PLoS ONE 6: e17288 doi:10.1371/journal.pone.0017288 21408061 10.1371/journal.pone.0017288 PMC3052304

[75] Aziz RK, Bartels D, Best AA, DeJongh M, Disz T, et al (2008) The RAST Server: rapid annotations using subsystems technology. BMC Genomics 9: 75 doi:10.1186/1471-2164-9-75 18261238 10.1186/1471-2164-9-75 PMC2265698

[76] Grissa I, Vergnaud G, Pourcel C (2007) CRISPRFinder: a web tool to identify clustered regularly interspaced short palindromic repeats. NAR 35: W52–W57 doi:10.1093/nar/gkm360 17537822 10.1093/nar/gkm360 PMC1933234

