# Supplementary figures and images for: Mechanistic Model of Rothia mucilaginosa Adaptation toward Persistence in the CF Lung, Based on a Genome Reconstructed from Metagenomic Data
Source: PLoS One. 2013 May 30;8(5):e64285. doi: 10.1371/journal.pone.0064285 (PMC3667864; doi:10.1371/journal.pone.0064285)

Base position in CF1E

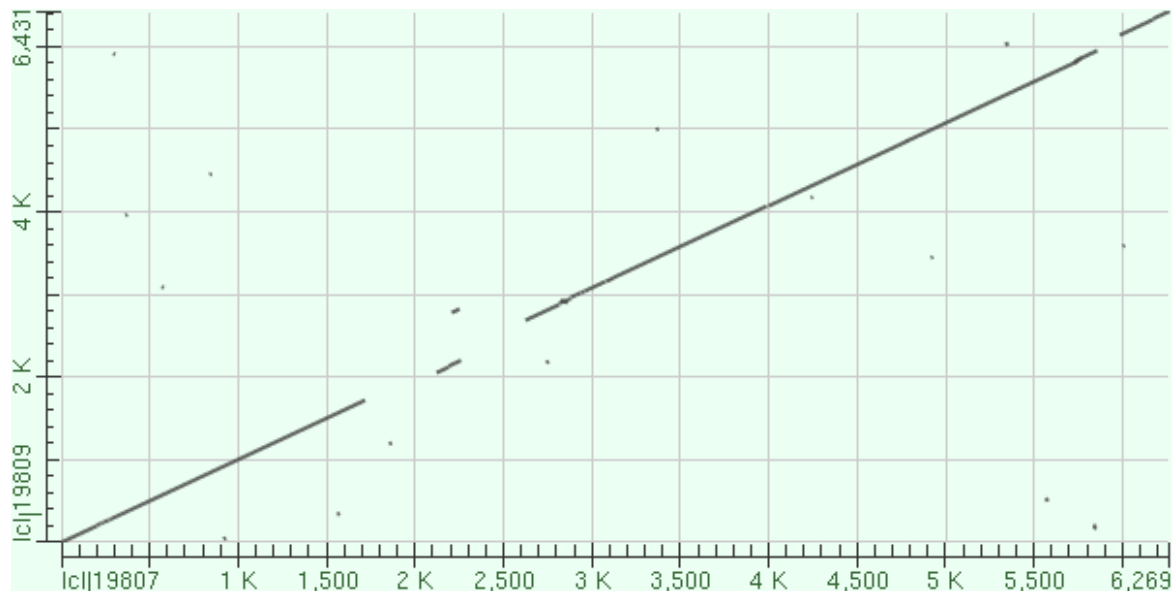

Base position in DY-18

Supplement: Figure S1 — Dot Plot matrix view of the alignment of CF1E type I restriction modification system (subunit M, R, S) against DY-18. (PDF) [file pone.0064285.s001.pdf]
