# Supplementary material for: Mechanistic Model of Rothia mucilaginosa Adaptation toward Persistence in the CF Lung, Based on a Genome Reconstructed from Metagenomic Data
Source: PLoS One. 2013 May 30;8(5):e64285. doi: 10.1371/journal.pone.0064285 (PMC3667864; doi:10.1371/journal.pone.0064285)
Supplement: Table S1 — Diseases associated with R. mucilaginosa. (PDF) [file pone.0064285.s002.pdf]

Disease	Reference(s)
Oral squamous cell carcinoma	(Pushalkar et al. 2011)
Periodontitis	(Siqueira Jr et al. 2007)
Branchial pouch anomalies	(Pahlavan et al. 2010)
Upper respiratory tract infection in sickle cell disease	(Rogovik et al. 2010)
Pneumonia	(Fusconi et al. 2009; Ko et al. 2009)
Endocarditis	(Pinsky et al. 1989)
Pericoronitis	(Peltroche-Llacsahttanga et al. 2000)
Psoriasis	(Gao et al. 2008)
Peritonitis	(Hodzic & Snyder 2010)
Bacteremia	(Kaufhold et al. 1992; Vaccher et al. 2007; Ohno et al. 2010)
Bacterial meningitis	(A. B. Lee et al. 2008)
Arthritis	(Kaasch et al. 2010)
Central nervous system infections	(Pulzova et al. 2009; Blouin et al. 2010)
Granulomatous dermatitis	(Morgan et al. 2010)
