# Supplementary material for: Mechanistic Model of Rothia mucilaginosa Adaptation toward Persistence in the CF Lung, Based on a Genome Reconstructed from Metagenomic Data
Source: PLoS One. 2013 May 30;8(5):e64285. doi: 10.1371/journal.pone.0064285 (PMC3667864; doi:10.1371/journal.pone.0064285)
Supplement: Table S2 — Microbiomes used in this study. Clinical status was designated as exacerbation (prior to systemic antibiotic treatment), on treatment (during systemic antibiotic treatment), post treatment (upon completion of systemic antibiotic treatment) or stable (when clinically stable and at their clinical and physiological baseline). The samples collected during exacerbation were designated as Day 0 sample, and the times between samples are cumulatively calculated from Day 0. (PDF) [file pone.0064285.s003.pdf]

Patient ID (Gender, Age)	Time Point	Time Line (Day)	Health Status
CF1 (Male, 38)	D	0	Exacerbation
	E	14	On Treatment
	F	33	Post Treatment
CF4 (Male, N/A)	A	0	Exacerbation
	B	11	Post Treatment
	C	58	Stable
CF5 (Female, N/A)	A	0	Exacerbation
	B	21	Post Treatment
CF6 (Female, 39)	A	0	Exacerbation
	B	12	On Treatment
	C	17	Post Treatment
	D	46	Stable
CF7 (Male, 36)	A	0	Exacerbation
	B	20	On Treatment
	C	27	On Treatment
	D	37	Post Treatment
CF8 (Male, 26)	A	0	Exacerbation
	B	17	Post Treatment
