# Supplementary material for: Mechanistic Model of Rothia mucilaginosa Adaptation toward Persistence in the CF Lung, Based on a Genome Reconstructed from Metagenomic Data
Source: PLoS One. 2013 May 30;8(5):e64285. doi: 10.1371/journal.pone.0064285 (PMC3667864; doi:10.1371/journal.pone.0064285)
Supplement: Table S3 — (A) Annotation of the gaps ≥5 kbp in CF1 metagenomic reference mapping against R. mucilaginosa DY-18. Refer to Table S2 for detailed patient samples information. (B) Annotation of the gaps ≥5 kbp in CF6 metagenomic reference mapping against R. mucilaginosa DY-18. Refer to Table S2 for detailed patient samples information. (C) Annotation of the gaps ≥5 kbp in CF7 metagenomic reference mapping against R. mucilaginosa DY-18. Refer to Table S2 for detailed patient samples information. (D) Annotation of the gaps ≥5 kbp in CF8 metagenomic reference mapping against R. mucilaginosa DY-18. Refer to Table S2 for detailed patient samples information. (PDF) [file pone.0064285.s004.pdf]

CF1	CF1-D-Ex (Day 0)		CF1-F-Pt (Day 33)		Genes
	Start	Stop	Start	Stop	
*	116,888	127,413	118,226	127,208	Seryl-tRNA synthetase (EC 6.1.1.11) CDS; Conserved protein with diacylglycerol kinase catalytic domain CDS; FIG01029139: hypothetical protein CDS; FIG01029400: hypothetical protein CDS; conserved hypothetical protein, putative cell filamentation protein CDS; putative cell filamentation protein CDS; hypothetical protein (6)
	191,241	198,554	192,364	198,337	FIG01028594: hypothetical protein CDS; FIG01029378: hypothetical protein CDS; FIG01028871: hypothetical protein CDS (2); hypothetical protein 2)
**	317,814	325,245	319,381	325,118	4 hypothetical proteins + ATP-binding protein of ABC transporter
	1,044,230	1,052,204	1,045,478	1,052,421	GTP pyrophosphokinase (EC 2.7.6.5), (p)ppGpp synthetase I CDS; Type I restriction-modification system, DNA-methyltransferase subunit M (EC 2.1.1.72) CDS; Type I restriction-modification system, specificity subunit S (EC 3.1.21.3) CDS; Type I restriction-modification system, restriction subunit R (EC 3.1.21.3) CDS
**	1,214,127	1,222,178	1,214,321	1,221,292	FIG01293855: hypothetical protein CDS; hypothetical protein (2)
	1,369,419	1,382,713	1,369,198	1,377,838	FIG01028573: hypothetical protein CDS; FIG01028929: hypothetical protein CDS; FIG01029243: hypothetical protein CDS; FIG01029252: hypothetical protein CDS; CRISPR-associated protein Cas1 CDS
*	1,784,753	1,798,606	1,786,942	1,798,038	FIG01028641: hypothetical protein CDS; Acetyltransferase (EC 2.3.1.-) CDS; FIG01028952: hypothetical protein CDS; glycosyl transferase, family 2 CDS; FIG01028912: hypothetical protein CDS; protein tyrosine phosphatase CDS; Ribose-phosphate pyrophosphokinase (EC 2.7.6.1) CDS; N-acetylglucosamine-1-phosphate uridyltransferase (EC 2.7.7.23) / Glucosamine-1-phosphate N-acetyltransferase (EC 2.3.1.157) CDS; ABC transporter, ATP-binding protein CDS; 4-diphosphocytidyl-2-C-methyl-D-erythritol kinase (EC 2.7.1.148) CDS
**	1,893,621	1,899,157	1,893,621	1,898,769	hypothetical protein (3); protein tyrosine phosphatase CDS
**	2,084,616	2,090,484	2,084,469	2,090,539	Amino acid ABC transporter, periplasmic amino acid-binding protein CDS; Cystathionine gamma-lyase (EC 4.4.1.1) CDS; O-acetylhomoserine sulfhydrylase (EC 2.5.1.49) CDS; hypothetical protein CDS

* Gaps present in CF1E

** Gaps present in CF1E with sizes slightly smaller than 5 kbp and therefore not shown in the line plots (Figure 2 and Supplementary Figure 1).

Table S3. cont

CF6	CF6-B-Tr (Day 12)		CF6-C-Pt (Day 17)		Genes
	Start	Stop	Start	Stop	
	293,008	301,901	293,008	299,553	NAD-dependent protein deacetylase of SIR2 family CDS; FIG01029042: hypothetical protein CDS (2); FIG01029042: hypothetical protein CDS; FIG01028880: hypothetical protein CDS; FIG01028880: hypothetical protein CDS
	1,045,416	1,056,255	1,045,555	1,056,118	Type I restriction-modification system, DNA-methyltransferase subunit M (EC 2.1.1.72) CDS; Type I restriction-modification system, specificity subunit S (EC 3.1.21.3) CDS; Type I restriction-modification system, restriction subunit R (EC 3.1.21.3) CDS; hypothetical protein (2)
	1,212,461	1,225,388	1,216,259	1,225,272	FIG01029335: hypothetical protein CDS; Triosephosphate isomerase (EC 5.3.1.1) CDS; FIG01293855: hypothetical protein CDS; hypothetical protein (2)
	1,236,551	1,241,615	1,236,259	1,242,067	FIG01293855: hypothetical protein CDS; FIG01293855: hypothetical protein CDS
	1,480,952	1,493,792	1,483,197	1,491,820	FIG01293855: hypothetical protein CDS; FIG01293855: hypothetical protein CDS; Glutamate-ammonia-ligase adenylyltransferase (EC 2.7.7.42) CDS; Glutamine synthetase type I (EC 6.3.1.2) CDS
	1,893,606	1,898,780	1,893,621	1,898,780	hypothetical protein (2)
	1,952,461	1,959,004	1,952,580	1,959,091	FIG01029071: hypothetical protein CDS; Cystathionine gamma-synthase (EC 2.5.1.48) CDS; Thymidylate kinase (EC 2.7.4.9) CDS; Thymidylate kinase (EC 2.7.4.9) CDS; Thymidylate kinase (EC 2.7.4.9) CDS; ACT domain protein CDS; Mannose-6-phosphate isomerase (EC 5.3.1.8) CDS
	2,160,027	2,187,871	2,160,027	2,187,948	Cell division protein FtsK CDS; Cell division protein FtsK CDS; FIG01029055: hypothetical protein CDS; hypothetical protein (14)

Table S3. cont

CF7	CF7-A-Ex (Day 0)		CF7-B-Tr (Day 20)		Genes
	Start	Stop	Start	Stop	
	119,183	130,754	119,330	130,480	Mainly non-coding region + 1 hypothetical protein
	317,552	324,682	319,276	325,352	non-coding region, deleted from the cf1e scaffold too
	815,783	821,768	812,145	818,057	putative two-component system response regulator CDS; FIG01029073: hypothetical protein CDS; FIG01029073: hypothetical protein CDS; FIG01029073: hypothetical protein CDS
	1,046,072	1,058,265	1,044,326	1,059,604	Type I restriction-modification system, DNA-methyltransferase subunit M (EC 2.1.1.72) CDS; Type I restriction-modification system, specificity subunit S (EC 3.1.21.3) CDS; Type I restriction-modification system, restriction subunit R (EC 3.1.21.3) CDS; hypothetical protein (4)
	1,213,573	1,227,654	1,216,352	1,221,526	non-coding region + half of hypothetical protein
	1,366,206	1,382,817	1,366,502	1,382,793	FIG01029408: hypothetical protein CDS; FIG01029391: hypothetical protein CDS; FIG01028573: hypothetical protein CDS; FIG01028929: hypothetical protein CDS; FIG01029243: hypothetical protein CDS; FIG01029252: hypothetical protein CDS; CRISPR-associated protein Cas1 CDS
	1,786,873	1,797,870	1,786,975	1,792,449	glycosyl transferase, family 2 CDS; FIG01028912: hypothetical protein CDS; protein tyrosine phosphatase CDS; Ribose-phosphate pyrophosphokinase (EC 2.7.6.1) CDS
			1,792,870	1,799,197	N-acetylglucosamine-1-phosphate uridyltransferase (EC 2.7.7.23) / Glucosamine-1-phosphate N-acetyltransferase (EC 2.3.1.157) CDS; ABC transporter, ATP-binding protein CDS; 4-diphosphocytidyl-2-C-methyl-D-erythritol kinase (EC 2.7.1.148) CDS; Dimethyladenosine transferase (EC 2.1.1.-) CDS
	1,888,835	1,898,868	1,892,504	1,898,840	FIG01028568: hypothetical protein CDS; hypothetical protein (2)
	1,971,322	1,988,862	1,972,818	1,982,106	Probable ATP-dependent helicase lhr (EC 3.6.1.-) CDS; FIG01028989: hypothetical protein CDS; Ribosomal large subunit pseudouridine synthase A (EC 4.2.1.70) CDS; putative glycosyl hydrolase CDS; putative glycosyl hydrolase CDS
	2,160,027	2,165,406	2,160,373	2,166,081	hypothetical protein (7)

Table S3. cont

CF8	CF8-A-Ex (Day 0)		CF8-B-Pt (Day 17)		Genes
	Start	Stop	Start	Stop	
	118,031	124,798	120,245	125,303	Non-coding region
	1,048,704	1,054,082			subunit R only that's missing
			1,045,578	1,054,259	Type I restriction-modification system, DNA-methyltransferase subunit M (EC 2.1.1.72) CDS; Type I restriction-modification system, specificity subunit S (EC 3.1.21.3) CDS; Type I restriction-modification system, restriction subunit R (EC 3.1.21.3) CDS; hypothetical protein (4)
	1,339,023	1,346,126			predicted nucleic acid-binding protein CDS; predicted nucleic acid-binding protein CDS; FIG01029207: hypothetical protein CDS;
			1,341,734	1,348,705	FIG01029207: hypothetical protein CDS; gluconolactonase CDS; protein of unknown function DUF34 CDS; Peptide methionine sulfoxide reductase MsrA (EC 1.8.4.11) CDS
	1,365,871	1,382,363	1,366,786	1,383,290	FIG01029408: hypothetical protein CDS; FIG01029391: hypothetical protein CDS; FIG01028573: hypothetical protein CDS; FIG01028929: hypothetical protein CDS; FIG01029243: hypothetical protein CDS; FIG01029252: hypothetical protein CDS; CRISPR-associated protein Cas1 CDS
	1,701,452	1,706,504	1,701,544	1,706,768	ABC transporter related CDS; ABC transporter related CDS; ABC transporter related CDS; ABC transporter related CDS; Protease II (EC 3.4.21.83) CDS
	1,893,711	1,898,815	1,893,481	1,898,769	FIG01028568: hypothetical protein CDS; protein tyrosine phosphatase CDS; protein tyrosine phosphatase CDS; hypothetical protein (2)
	1,971,500	1,981,982	1,975,975	1,981,085	Probable ATP-dependent helicase lhr (EC 3.6.1.-) CDS; FIG01028989: hypothetical protein CDS; Ribosomal large subunit pseudouridine synthase A (EC 4.2.1.70) CDS; putative glycosyl hydrolase CDS
