# Supplementary material for: Mechanistic Model of Rothia mucilaginosa Adaptation toward Persistence in the CF Lung, Based on a Genome Reconstructed from Metagenomic Data
Source: PLoS One. 2013 May 30;8(5):e64285. doi: 10.1371/journal.pone.0064285 (PMC3667864; doi:10.1371/journal.pone.0064285)
Supplement: Table S4 — Statistics from BWA mapping of metagenomic reads against the reference genome R. mucilaginosa DY-18. (PDF) [file pone.0064285.s005.pdf]

Metagenome	Number of Mapped Reads	Maximum Coverage	Mean Coverage	Number of Bases (% bases of reference genome)
CF1-D	4,772	12	0.9	1,239,444 (55%)
CF1-E	41,835	47	7.8	2,117,375 (93%)
CF1-F	12,512	17	2.4	1,892,620 (84%)
CF4-C	8,886	23	1.6	1,621,081 (72%)
CF6-A	355	6	0.06	131,287 (6%)
CF6-B	9,925	18	1.8	1,683,390 (74%)
CF6-C	10,790	20	1.9	1,728,332 (76%)
CF6-D	800	8	0.15	285,337 (13%)
CF7-A	5,088	16	0.9	1,220,642 (54%)
CF7-B	9,588	16	1.7	1,659,075 (73%)
CF7-C	133	7	0.02	42,330 (2%)
CF7-D	1,246	8	0.2	416,645 (18%)
CF8-A	10,225	50	1.8	1,6,45,293 (73%)
CF8-B	11,236	16	2.0	1,783,108 (79%)
