# Supplementary material for: Mechanistic Model of Rothia mucilaginosa Adaptation toward Persistence in the CF Lung, Based on a Genome Reconstructed from Metagenomic Data
Source: PLoS One. 2013 May 30;8(5):e64285. doi: 10.1371/journal.pone.0064285 (PMC3667864; doi:10.1371/journal.pone.0064285)
Supplement: Table S5 — General features of the CF1E R. mucilaginosa scaffold, DY-18 reference genome, and M508 draft genome. (PDF) [file pone.0064285.s006.pdf]

Rothia mucilaginosa	**CF1E**	**DY-18 (reference)**	**M508***
**Genome Size**	2,278,618 bp	2,264,603 bp	2,313,271
**GC content**	59.6%	59.6%	59.6%
**Predicted gene products****	1,739	1,739	1,790
**Total subsystems** (%)**	248 (42%)	254 (42%)	251 (41%)

* Draft genome in supercontigs format from the Genomes Online Database (GOLD).

** Numbers obtained from the RAST-annotation server
