# Supplementary material for: Mechanistic Model of Rothia mucilaginosa Adaptation toward Persistence in the CF Lung, Based on a Genome Reconstructed from Metagenomic Data
Source: PLoS One. 2013 May 30;8(5):e64285. doi: 10.1371/journal.pone.0064285 (PMC3667864; doi:10.1371/journal.pone.0064285)
Supplement: Table S6 — Subsystem feature counts of R. mucilaginosa CF1E, DY-18, and M508. (PDF) [file pone.0064285.s007.pdf]

Subsystem features	CF1E	DY-18	M508
Amino Acids and Derivatives	153	162	148
Protein Metabolism	142	142	141
Cofactors, Vitamins, Prosthetic Groups, Pigments	136	130	128
Carbohydrates	101	101	107
RNA Metabolism	90	89	93
DNA Metabolism	63	81	79
Nucleosides and Nucleotides	59	60	60
Cell Wall and Capsule	58	60	57
Stress Response	50	50	50
Fatty Acids, Lipids, and Isoprenoids	29	29	29
Virulence, Disease and Defense	25	22	24
Respiration	22	21	22
Membrane Transport	18	18	17
Cell Division and Cell Cycle	13	14	14
Phosphorus Metabolism	13	13	13
Regulation and Cell signaling	10	10	10
Nitrogen Metabolism	9	9	9
Potassium metabolism	9	9	9
Iron acquisition and metabolism	7	7	4
Sulfur Metabolism	5	5	6
Dormancy and Sporulation	1	1	1
Metabolism of Aromatic Compounds	1	1	1
Miscellaneous	50	50	49

Subsystem features that do not have any count: photosynthesis; Phages, Prophages, Transposable elements, Plasmids; motility and chemotaxis; secondary metabolism.
