# Supplementary material for: Mechanistic Model of Rothia mucilaginosa Adaptation toward Persistence in the CF Lung, Based on a Genome Reconstructed from Metagenomic Data
Source: PLoS One. 2013 May 30;8(5):e64285. doi: 10.1371/journal.pone.0064285 (PMC3667864; doi:10.1371/journal.pone.0064285)
Supplement: Table S7 — Sequence identities of the genes encoding the Type I restriction modification system in CF1E and DY-18, determined using BLAST. The identity value is subjected to >97% query length coverage. (PDF) [file pone.0064285.s008.pdf]

	Nucleotide level Identity against DY-18 (%)	Protein level Identity against DY-18 (%)
**Subunit R**	81	75
**Subunit M**	87	88
**Subunit S**	37	41
