# Supplementary material for: Mechanistic Model of Rothia mucilaginosa Adaptation toward Persistence in the CF Lung, Based on a Genome Reconstructed from Metagenomic Data
Source: PLoS One. 2013 May 30;8(5):e64285. doi: 10.1371/journal.pone.0064285 (PMC3667864; doi:10.1371/journal.pone.0064285)
Supplement: Table S8 — CRISPR positions in the CF1E genome scaffold. (PDF) [file pone.0064285.s009.pdf]

start	end	length	DR length	No. of Spacers	Blastx against NR
1,389,037	1,389,864	827	36	11	hypothetical protein from *Caenorhabditis remanei*
1,392,263	1,393,579	1,316	36	17	no hit
1,394,037	1,395,297	1,260	36	17	hypothetical protein from *Propionibacterium freudenreichii* subsp. Shermanii CIRM-BIA1
1,395,433	1,395,686	253	36	3	no hit
