# Supplementary material for: Mechanistic Model of Rothia mucilaginosa Adaptation toward Persistence in the CF Lung, Based on a Genome Reconstructed from Metagenomic Data
Source: PLoS One. 2013 May 30;8(5):e64285. doi: 10.1371/journal.pone.0064285 (PMC3667864; doi:10.1371/journal.pone.0064285)
Supplement: Table S9 — Identification of the spacer sequences in CF1E CRISPR structure from human- and environmental-viral metagenomes at 100% length coverage and ≥90% identity (≤2 mismatches). (PDF) [file pone.0064285.s010.pdf]

Virome (Habitat)	Spacer sequence	No. of hits
(Willner et al. 2011) **(Oral)**<br><br>5b11623defc42938f41589107350896f_186762_266<br>5b11623defc42938f41589107350896f_176283_256<br>5b11623defc42938f41589107350896f_176158_244<br>5b11623defc42938f41589107350896f_175980_260<br>5b11623defc42938f41589107350896f_175807_233<br>5b11623defc42938f41589107350896f_173039_268<br>5b11623defc42938f41589107350896f_159634_255<br>5b11623defc42938f41589107350896f_159104_258<br>5b11623defc42938f41589107350896f_158624_261<br>5b11623defc42938f41589107350896f_118402_230<br>5b11623defc42938f41589107350896f_84294_248<br>5b11623defc42938f41589107350896f_70709_261<br>5b11623defc42938f41589107350896f_60807_263<br>5b11623defc42938f41589107350896f_60398_267<br>5b11623defc42938f41589107350896f_58332_260<br>5b11623defc42938f41589107350896f_48083_250<br>5b11623defc42938f41589107350896f_37476_262<br>5b11623defc42938f41589107350896f_33576_249<br>5b11623defc42938f41589107350896f_18007_255	CAACGATTCCCACGCGGCGCGCCCAGTCTCCGTCTGA	**19**
(Willner et al. 2011) **(Oral)**<br><br>f44d959b723905a049b0334f19668e5c_207520_157<br>f44d959b723905a049b0334f19668e5c_201888_260<br>f44d959b723905a049b0334f19668e5c_110173_251<br>f44d959b723905a049b0334f19668e5c_212940_122	CAACGATTCCCACGCGGCGCGCCCAGTCTCCGTCTGA	**4**
