# Supplementary material for: Mechanistic Model of Rothia mucilaginosa Adaptation toward Persistence in the CF Lung, Based on a Genome Reconstructed from Metagenomic Data
Source: PLoS One. 2013 May 30;8(5):e64285. doi: 10.1371/journal.pone.0064285 (PMC3667864; doi:10.1371/journal.pone.0064285)
Supplement: Table S10 — A comparison of putative adaptations and predicted metabolisms of R. mucilaginosa and P. aeruginosa that are hypothesized to enable persistence in the CF lung, based on literature and genomic data. (PDF) [file pone.0064285.s011.pdf]

**CF-lung adapted phenotype**	***Pseudomonas aeruginosa ****	***Rothia mucilaginosa***
**Respiration**	Undergoes aerobic, microaerobic, & anaerobic respiration<br>Increasing denitrification<br>Fermentation	Aerobic<br>Microaerobic<br>Anaerobic (very slow growing)<br>Reduces nitrate<br>Fermentation
**Food source**	Free amino acids<br>(Prefers L-alanine, L-arginine, L-glutamate)<br>Lactate<br>Pyruvate<br>Arginine	Free amino acids<br>Sucrose<br>Fructose<br>Lactate<br>Glycerol & glycerol-3-phosphate<br>Pyruvate
**Motility**	Uses flagella but this is lost when *P. aeruginosa* adapts towards persistence	Not known
**Mucoidy / Biofilm**	Conversion to mucoidy (overproduction of alginate) in persistent infection<br>Biofilm production is alginate – dependent	Organism contains mucoid capsule<br><br>Biofilm production is mannose-dependent
**Signaling and communication systems**	Loss of quorum sensing molecules e.g. AHLs due to ΔLasR<br>*Rhs* may be responsible for the communication between self, competitors & host?	No known quorum sensing molecules<br><br>*Rhs* may be responsible for the *communication* between self, competitors & host?
**Type III secretion**	Down-regulated or lost to reduce virulence	Not detected in the genome
**Other virulence factors**	Loss of virulence including secretion of elastase and exotoxin	-
**Siderophores**	Pyoverdine and pyochelin	Not detected in the genome
**Iron acquisition**	Multiple mechanisms; independent systems for heme, $Fe3+$ and $Fe2+$ uptake and use	Heme, hemin uptake and utilization systems in Gram Positives; Low-pH induced ferrous iron transporter
**Antibiotic resistance mechanisms**	β-lactamases<br>Mex – multidrug efflux pumps<br>Loss of OprD (Imipenem resistance)<br>Mutation – induced	Modulator of Drug Activity (MdaB)<br>Macrolide export ATP-binding/permease protein MacB<br>EmrB/QacA subfamily drug resistance transporter<br>Bcr/CflA family multidrug resistance transporter
**Prophages**	Detected in the genome	Not detected in the genome

* Mainly extracted from (Hogardt & Heesemann 2010)
