# Supplementary material for: Mechanistic Model of Rothia mucilaginosa Adaptation toward Persistence in the CF Lung, Based on a Genome Reconstructed from Metagenomic Data
Source: PLoS One. 2013 May 30;8(5):e64285. doi: 10.1371/journal.pone.0064285 (PMC3667864; doi:10.1371/journal.pone.0064285)
Supplement: Table S11 — Genes that are missing from the CF1E genome scaffold but present in the DY-18 reference. Genes are considered missing when the gap is within a contig. (PDF) [file pone.0064285.s012.pdf]

Name	Length	Start	End	Region Size
predicted ARSR subfamily of helix-turn-helix bacterial transcription regulatory protein CDS	396	43502	44199	697
conserved hypothetical protein, putative cell filamentation protein CDS	693	119455	127207	7752
FIG01029139: hypothetical protein CDS	687	119455	127207	7752
FIG01029400: hypothetical protein CDS	756	119455	127207	7752
hypothetical protein CDS	210	119455	127207	7752
hypothetical protein CDS	156	119455	127207	7752
hypothetical protein CDS	210	119455	127207	7752
hypothetical protein CDS	189	119455	127207	7752
hypothetical protein CDS	561	119455	127207	7752
hypothetical protein CDS	285	119455	127207	7752
putative cell filamentation protein CDS	636	119455	127207	7752
exopolysaccharide biosynthesis protein related to N-acetylglucosamine-1-phosphodiester alpha-N-acetylglucosaminidase CDS	1317	138835	139305	470
predicted nucleic acid-binding protein CDS	489	138835	139305	470
UBA/THIF-type NAD/FAD binding fold CDS	1071	149644	150934	1290
hypothetical protein CDS	1179	260142	261857	1715
hypothetical protein CDS	117	260142	261857	1715
hypothetical protein CDS	1314	283582	285106	1524
ATP-binding protein of ABC transporter CDS	777	319311	324416	5105
FIG01028874: hypothetical protein CDS	1215	319311	324416	5105
FIG01029167: hypothetical protein CDS	792	319311	324416	5105
hypothetical protein CDS	333	319311	324416	5105
hypothetical protein CDS	903	319311	324416	5105
ABC-type amino acid transport system, permease component CDS	684	620953	624517	3564
COG1126: ABC-type polar amino acid transport system, ATPase component CDS	765	620953	624517	3564
Glutamate binding protein CDS	831	620953	624517	3564
putative glutamate transporter permease protein CDS	939	620953	624517	3564
FIG01029195: hypothetical protein CDS	1194	656126	657389	1263
hypothetical protein CDS	1791	1237261	1239756	2495
putative integral membrane protein CDS	687	1410305	1411220	915
Pyruvate oxidase [ubiquinone, cytochrome] (EC 1.2.2.2) CDS	1752	1817923	1820007	2084
cell wall surface anchor family protein CDS	735	1924698	1925692	994
Amino acid ABC transporter, periplasmic amino acid-binding protein CDS	846	2084618	2090544	5926
Cystathionine gamma-lyase (EC 4.4.1.1) CDS	1161	2084618	2090544	5926
hypothetical protein CDS	1029	2084618	2090544	5926
O-acetylhomoserine sulfhydrylase (EC 2.5.1.49) CDS	1152	2084618	2090544	5926
FIG01029286: hypothetical protein CDS	981	2105030	2107749	2719
FIG01029131: hypothetical protein CDS	1476	2209635	2211353	1718
