# Supplementary material for: Mechanistic Model of Rothia mucilaginosa Adaptation toward Persistence in the CF Lung, Based on a Genome Reconstructed from Metagenomic Data
Source: PLoS One. 2013 May 30;8(5):e64285. doi: 10.1371/journal.pone.0064285 (PMC3667864; doi:10.1371/journal.pone.0064285)
Supplement: Table S12 — Genes present in the CF1E genome scaffold but missing in the reference genome DY-18. (PDF) [file pone.0064285.s013.pdf]

Name	Insertion site	region size
hypothetical protein CDS<br>Phage lysin, N-acetylmuramoyl-L-alanine amidase CDS	53748	3174
Modulator of drug activity B CDS	65967	913
Putative DNA-binding protein CDS	70542	1534
hypothetical protein CDS	92872	1160
hypothetical protein CDS	111545	710
hypothetical protein CDS	119455	1338
predicted nucleic acid-binding protein CDS<br>hypothetical protein CDS	156926	1615
putative hydrolase CDS	239949	1690
hypothetical protein CDS	374555	1065
acyltransferase 3 CDS	613830	2685
hypothetical protein CDS	625770	945
hypothetical protein CDS	686303	719
Mobile element protein CDS	808,821	318
hypothetical protein CDS<br>DNA-cytosine methyltransferase (EC 2.1.1.37) CDS<br>hypothetical protein CDS	992646	3146
2-oxoglutarate/malate translocator CDS	1013016	1866
putative helicase CDS<br>putative helicase CDS<br>hypothetical protein CDS	1084123	5306
FIG01029049: hypothetical protein CDS<br>hypothetical protein CDS	1383788	1489
hypothetical protein CDS	1392429	754
PE-PGRS FAMILY PROTEIN CDS	1434185	4251
hypothetical protein CDS	1464431	1830
Mercuric ion reductase (EC 1.16.1.1) CDS	1671908	1271
Macrolide export ATP-binding/permease protein MacB (EC 3.6.3.-) CDS<br>hypothetical protein CDS	1671564	3475
hypothetical protein CDS<br>hypothetical protein CDS	1788171	2192
hypothetical protein CDS<br>hypothetical protein CDS	1790071	2205
hypothetical protein CDS	1817923	1800
Mobile element protein CDS	1884458	1840
L-lactate dehydrogenase (EC 1.1.2.3) CDS	2044067	1547
Flagellar hook-length control protein fliK CDS	1924657	966
hypothetical protein CDS	2000796	1310
