# Supplementary material for: Mechanistic Model of Rothia mucilaginosa Adaptation toward Persistence in the CF Lung, Based on a Genome Reconstructed from Metagenomic Data
Source: PLoS One. 2013 May 30;8(5):e64285. doi: 10.1371/journal.pone.0064285 (PMC3667864; doi:10.1371/journal.pone.0064285)
Supplement: Table S13 — Isolation source and references of sequences extracted and used in the 16S phylogenetic analysis. (PDF) [file pone.0064285.s014.pdf]

Category	Organism (Accession)	Isolation source	Underlying disease	Reference
Human	*Rothia arfidiae* SMC-A6087 (DQ673322)	Blood	Pneumonia	Ko KS et al. (2009)
	Rothia mucilaginosa CF1E	Sputum from CF	Cystic Fibrosis	This Paper (2012)
	Rothia mucilaginosa DY-18 (NC_013715)	Oral	Persistent apical periodontitis lesion	Yamane K. et al. (2010)
	Rothia sp. Lab 21-1_x2 (GQ900877)	Sputum from CF	Cystic Fibrosis	Guss AM. et al. (2011)
	Rothia sp. sp2-iso-om10x3 (GQ900840)	Sputum from CF	Cystic Fibrosis	Guss AM. et al. (2011)
	Rothia mucilaginosa ATCC 25296	Oral	-	HMP unpublished
	Rothia sp. sp3-iso-117x2 (GQ900845)	Sputum from CF	Cystic Fibrosis	Guss AM. et al. (2011)
	Rothia sp. ChDC B201 (AF543279)	Oral	-	unpublished
	Rothia sp. Smarlab 3302411 (AY538697)	Bronchic expectoration	-	unpublished
	Rothia sp. Sp3-iso-110x2 (GQ900846)	Sputum from CF		Guss AM. et al. (2011)
	Rothia sp. Oral taxon 188 (GU470892)	Oral	-	Dewhirst FE. (2010)
	Rothia sp. sp2-iso-AG4x3 (GQ900837)	Sputum from CF	-	Guss AM. et al. (2011)
	Rothia dentocariosa ATCC 17931 (CP002280)	Oral	-	HMP unpublished
Environmental	*Rothia sp.* CMG M10 (EU081515)	Pakistan coastline	-	Uzair B. (Ref unclear)
	Rothia sp.YIM C456 (EU135638)	Haloalkaline soil	-	Cui XL. et al. (Ref unclear)
	*Rothia sp.*ZHT413 (EU873349)	Venerupis philippinaram shell conglutination mud	-	unpublished
	Rothia sp. JSM 078151	Saline soil	-	unpublished
	Rothia sp. RA22 (FJ898305)	spring	-	Yang H and Lou K. (2011)
	Rothia sp. ZF11 (GQ891672)	water from high level natural radiation area	-	Zakei D. et al. (2010)
	Rothia sp. LH-CAB6 (HQ717389)	Air around 4200 m	-	unpublished
	Rothia sp. DG3 (JN208194)	Soil	-	unpublished
	Rothia sp. BBH4 (AM183255)	Deep sea sediment	-	unpublished
	Rothia amarae (AY043359)	Foul water sewer sludge	-	Fan Y. et al. (2002)

	Rothia sp. RV13 (GU318366)	Dysidea tupha (marine sponge)	-	unpublished
	Rothia sp. 3_1/4V	Semi-coke	-	unpublished
	Rothia terrae strain L-143 (NR_043968)	soil	-	Chou YJ. et al. (2008)
	Rothia sp. J03 (DQ409139)	Herbs in tumulus	-	unpublished
	Rothia sp. Piab1P (DQ457602)	nodules of Hedysarum glomeratum	-	Muresu r. et al. (2008)
Milk	*Rothia sp.* H29 (EF204383)	Raw milk	-	Hantsis-Zacharov E. et al. (2007)
	Rothia sp. H7 (EF204384)	Raw milk	-	Hantsis-Zacharov E. et al. (2007)
	Rothia sp. H21 (EF204385)	Raw milk	-	Hantsis-Zacharov E. et al. (2007)
Cheese	*Rothia sp.* R-23177 (AJ969174)	Smear-ripened cheese	-	unpublished
Animal	*Rothia sp.* C158-P CA-T3P21			
	Rothia sp. BP (EU725780)	Poultry - India	Otitis	unpublished
	Rothia sp. CCUG 25688 (AJ131122)	Mouse nose	-	Collins et al. (2000)
Ourgroup	*Escherichia coli* str. K-12 substr. MG1655 (NC_000913)			
	Pseudomonas aeruginosa PAO1 (NC_002516)			
