# Supplementary material for: Mechanistic Model of Rothia mucilaginosa Adaptation toward Persistence in the CF Lung, Based on a Genome Reconstructed from Metagenomic Data
Source: PLoS One. 2013 May 30;8(5):e64285. doi: 10.1371/journal.pone.0064285 (PMC3667864; doi:10.1371/journal.pone.0064285)
Supplement: Table S14 — Protein-coding genes used for multilocus phylogenetic inference. (PDF) [file pone.0064285.s015.pdf]

**Organism**	**Isolation Source**	**Genes (Accession)**
Rothia dentocariosa ATCC 17931	Human oral	RecA (ADP41036)<br>RpoA (ADP39732)<br>Inf2 (ADP41005)
Rothia dentocariosa M567	Human oral cavity	RecA (EFJ76920)<br>RpoA (EFJ77777)<br>Inf2 (ZP_07072987)
Rothia mucilaginosa ATCC 25296	Human oral	RecA (EET75911)<br>RpoA (EET75429)<br>Inf2 (ZP_05367468)
Rothia mucilaginosa DY-18	Human oral	RecA (BAI65285)<br>RpoA (BAI64339)<br>Inf2 (BAI65313)
Rothia mucilaginosa M508	Human airways sample	RecA (EHB87709)<br>RpoA (EHB88703)<br>Inf2 (EHB87687)
Rothia mucilaginosa CF1E	Cystic Fibrosis sputum	RecA<br>RpoA (This study)<br>Inf2
