# Supplementary material for: Mechanistic Model of Rothia mucilaginosa Adaptation toward Persistence in the CF Lung, Based on a Genome Reconstructed from Metagenomic Data
Source: PLoS One. 2013 May 30;8(5):e64285. doi: 10.1371/journal.pone.0064285 (PMC3667864; doi:10.1371/journal.pone.0064285)
Supplement: Table S15 — Genes missing from the CF1E genome scaffold, based on contig mapping to the reference genome DY18. (PDF) [file pone.0064285.s016.pdf]

Gene name	Gene length
1-acyl-sn-glycerol-3-phosphate acyltransferase (EC 2.3.1.51) CDS	681
Cell division protein FtsI [Peptidoglycan synthetase] (EC 2.4.1.129) CDS	1,866
COG0834: ABC-type amino acid transport/signal transduction systems, periplasmic component/domain CDS	840
COG4420: Predicted membrane protein CDS	933
Dihydrolipoamide dehydrogenase (EC 1.8.1.4) CDS	1,407
FIG01028708: hypothetical protein CDS	1,464
FIG01029080: hypothetical protein CDS	153
FIG01029402: hypothetical protein CDS	1,749
FIG01114456: hypothetical protein CDS	1,146
PTS system, mannose-specific IIC component (EC 2.7.1.69) CDS	867
putative ABC transporter ATP-binding protein CDS	114
Ribonuclease D (EC 3.1.26.3) CDS	1,266
Transcriptional regulator, FUR family CDS	591
