# Supplementary material for: Mechanistic Model of Rothia mucilaginosa Adaptation toward Persistence in the CF Lung, Based on a Genome Reconstructed from Metagenomic Data
Source: PLoS One. 2013 May 30;8(5):e64285. doi: 10.1371/journal.pone.0064285 (PMC3667864; doi:10.1371/journal.pone.0064285)
Supplement: Supporting Information S1 — Additional samples information. (DOCX) [file pone.0064285.s017.docx]

**Supporting Information S1**

Samples information

The patients were selected based on the eligibility criteria that include (i) a known clinical diagnosis of CF, (ii) a protocol defined exacerbation that requires intravenous antibiotics, and (iii) a drop in FEV_1_ of at least 15% or more compared to the best FEV_1_ in the past 12 months. Sputum samples were collected from six CF volunteers (Table S2) at the Adult CF Clinic (San Diego, CA, United States) by expectoration into a sterile cup except sample CF4-A that was a tracheal aspirate. All collection was in accordance with the University of California Institutional Review Board (HRPP 081500) and San Diego State University Institutional Review Board (SDSU IRB#2121). Clinical status at the time of collection was designated as *exacerbation* (prior to systemic antibiotic treatment), *on* *treatment* (during systemic antibiotic treatment), *post treatment* (upon completion of systemic antibiotic treatment) or *stable* (when clinically stable and at their clinical and physiological baseline). The samples collected during exacerbation were designated as Day 0 sample.
