# Supplementary material for: Mechanistic Model of Rothia mucilaginosa Adaptation toward Persistence in the CF Lung, Based on a Genome Reconstructed from Metagenomic Data
Source: PLoS One. 2013 May 30;8(5):e64285. doi: 10.1371/journal.pone.0064285 (PMC3667864; doi:10.1371/journal.pone.0064285)
Supplement: Supporting Information S2 — Rothia mucilaginosa in cystic fibrosis community metatranscriptomes. (DOCX) [file pone.0064285.s018.docx]

**Supporting Information S2**

*R. mucilaginosa* in community metatranscriptomes

*Rothia* was detected in 3/5 metatranscriptomes (Supplementary Figure D1). Even though *R. mucilaginosa* was present in high abundance in the CF1 samples, the number of metatranscriptomic sequences in these samples was too small for significant detection. Metatranscriptome was not generated for the CF1E sample. On the other hand, CF4C, the only time point from patient CF4 containing *R. mucilaginosa* (~20% in the microbiome), had 120 *R. mucilaginosa* hits to mRNAs (1.6% of microbial transcripts) and 15 hits to rRNAs (0.2% of microbial transcripts) in its metatranscriptome. The transcripts were scattered randomly across the genome (Supplementary Figure D2) starting from position 148,000 (no coverage was detected before this position).

| Sample | Total number of sequences | Total number of non-rRNA reads | Total number of microbial transcripts | Total  *R. mucilaginosa* hits (rRNA) | Total  *R. mucilaginosa* hits (non-rRNA) |
| --- | --- | --- | --- | --- | --- |
| CF1D | 1,991 | 1,900 | 283 | 4 | 0 |
| CF1F | 25,238 | 7,971 | 312 | 3 | 2 |
| CF4A | 68,414 | 59,394 | 1,030 | 0 | 0 |
| CF4B | 32,737 | 32,446 | 471 | 0 | 0 |
| CF4C | 36,172 | 34,411 | 7,442 | 15 | 120 |

* No metatranscriptome was generated from CF1E

SI Fig. D1: The prevalence of *R. mucilaginosa* in the corresponding community transcriptomes.

SI Fig. D2: Coverage of 120 CF4C metatranscriptomic mRNA reads on the reference genome *R. mucilaginosa* DY-18 starting from position 148,000 bp.
