# Supplementary material for: Mechanistic Model of Rothia mucilaginosa Adaptation toward Persistence in the CF Lung, Based on a Genome Reconstructed from Metagenomic Data
Source: PLoS One. 2013 May 30;8(5):e64285. doi: 10.1371/journal.pone.0064285 (PMC3667864; doi:10.1371/journal.pone.0064285)
Supplement: Supporting Information S3 — Additional genome characteristic of R. mucilaginosa and phylogenetic analysis of Rothia spp. associated with cystic fibrosis. (DOCX) [file pone.0064285.s019.docx]

**Supporting Information S3**

**Characteristics of CF1E**

1. **Defining Gaps**

Gaps in the genome scaffold are defined as regions present in the reference DY-18 genome that were not covered by contigs in the final scaffold. The size of each gap was based on the difference in scaffolding coordinates at the end of the previous contig and the start of the next contig. For building the scaffold, gapped regions were filled with the ambiguous base N. There were only four gaps spanning more than 1 kbp, and in the reference genome these regions contained genes with unknown functions (i.e., hypothetical proteins). The genes present in the reference genome that correspond to the missing regions in CF1E are listed in Table S15.

1. **Phylogenetic analysis of *Rothia* spp.**

All 16S rRNA gene sequences from *Rothia* spp (n=36) were identified and retrieved from the NCBI nucleotide database (Table S6) and aligned with a full-length 16S rRNA gene from the CF1E genome scaffold. The phylogenetic analysis of these 16S rRNA gene sequences revealed four distinct groups of human *Rothia*-isolates that were strongly supported in both Bayesian inferences (≥95% Bayesian posterior probability) and maximum likelihood-based resampling (bootstrap of ≥70%) (SI Fig. D2A). The closest neighbor of *R. mucilaginosa* CF1E was DY-18, followed by isolates of *Rothia* sp. from CF patients. In addition, the tree showed that other CF *Rothia* spp. isolates belong to either the *R. mucilaginosa* group or *R. dentocariosa* group. The two *Rothia* spp. that clustered with the human group were isolated from mouse nose (*Rothia* sp. CCUG 25688) and herbs in tumulus (*Rothia* sp. J03).

A phylogenetic analysis of *Rothia* species based on the RecA, RpoA, and Inf2 amino acid sequences placed CF1E closest to the ATCC strain 25296 instead of DY-18 (SI Fig. D2B). However, CF1E was placed in the *R. mucilaginosa* group with strong support according to both Bayesian posterior probability and maximum likelihood bootstrapping.

*Methods used for Phylogenetic analysis*

16S rRNA sequences of the genus *Rothia* were downloaded from GenBank and compared to the 16S fragment from assembled CF1E contig 0173. RecA, RpoA, and Inf2 protein sequences of *R. mucilaginosa* and *R. dentocariosa* were downloaded from GenBank (accession numbers in Table S13 and S14). Each gene was aligned separately using MUSCLE (version 3.8.31) [1] and the alignment was trimmed using trimAI (version v1.4.rev7) [2]. The three separate gene alignments were then combined into a single alignment. For each dataset (16S rRNA and protein-coding genes), a phylogeny was estimated using MrBayes (version 3.1.2) [3] by four independent runs with the GTR+I+Ґ model of evolution, sampling every 100 generations for 10 X 10^6^ generations. Support for nodes was assessed with maximum likelihood bootstrapping as implemented in RAxML (version 7.2.6) [4] with the GTRGAMMAI model for 16S rRNA sequences and the PROTGAMMADAYHOFF model for amino acid sequences, both using default parameters.

(A)

(B)

SI Fig. D2: Phylogeny of *Rothia* species, a majority-rule consensus phylogram computed from the set of 36,000 credible trees. (A) Phylogeny based on the 16S rDNA. A “+” above a node indicates a branch supported by ≥95% Bayesian posterior probability and a “++” indicates additional maximum likelihood bootstrap of ≥70%. Branches with double diagonal lines have been shortened 8-fold to aid viewing. (B) Multilocus phylogeny based on the RecA, RpoA, and Inf2 protein sequences. A “++” above a node indicates a branch supported by ≥95% Bayesian posterior probability and maximum likelihood bootstrap of ≥85%. Branches with double diagonal lines have been shortened by 10.4 times to aid viewing. Detailed accession number, source of isolation and reference for each sequence are shown in Supplementary Table 13 and 14.

SI Fig. D3: Majority-rule consensus phylogram of sequenced partial Actinobacteria-specific 16S PCR fragments from lung sections. The branch support is indicated by Bayesian posterior probability. Lung 7 corresponds to patient CF7 who microbiomes were generated before the lung transplant, and the Lung 9 patient was not used in our metagenomic analysis. Branches with double diagonal lines have been shortened by ~3-fold to aid viewing.

**References**

1. Edgar RC (2004) MUSCLE: Multiple Sequence Alignment with High Accuracy and High Throughput. Nucl Acids Res 32: 1792–1797. doi:10.1093/nar/gkh340.

2. Capella-Gutiérrez S, Silla-Martínez JM, Gabaldón T (2009) trimAl: A Tool for Automated Alignment Trimming in Large-Scale Phylogenetic Analyses. Bioinformatics 25: 1972–1973. doi:10.1093/bioinformatics/btp348.

3. Ronquist F, Huelsenbeck JP (2003) MrBayes 3: Bayesian Phylogenetic Inference Under Mixed Models. Bioinformatics 19: 1572–1574. doi:10.1093/bioinformatics/btg180.

4. Stamatakis A (2006) RAxML-VI-HPC: Maximum Likelihood-Based Phylogenetic Analyses with Thousands of Taxa and Mixed Models. Bioinformatics 22: 2688–2690. doi:10.1093/bioinformatics/btl446.
